# The Hydration of Trifluoroacetic Acid from 0 to 298 K

**DOI:** 10.1021/acs.jpca.5c08151

**Published:** 2026-01-20

**Authors:** Walker J. Smith, Caroline S. Glick, George C. Shields

**Affiliations:** Department of Chemistry, 3628 Furman University, 3300 Poinsett Hwy, Greenville, South Carolina 29613, United States

## Abstract

Trifluoroacetic acid (TFA), the most atmospherically abundant perfluorocarbocylic acid, is a molecule of increasing environmental and biological significance. In this paper, we examine TFA’s role in new particle formation (NPF), a critical yet lesser-understood step in cloud formation in which aerosols that act as cloud condensation nuclei are formed. We used conformational sampling to find low-energy structures for TFA-*n*H_2_O (*n* = 1–8) clusters and determined accurate DLPNO–CCSD­(T)/haug-cc-pV5Z//ωB97X-D/6–31++G** enthalpies at 0 K and Gibbs free energies at 216.65, 273.15, and 298.15 K. Then, atmospheric concentrations were determined at relevant atmospheric temperatures. Rotational constants for the lowest energy *n* = 1–3 structures corroborate existing microwave spectroscopic data, validating our methodology. Additionally, spectroscopic properties for *n* = 4–8 structures were identified for comparison with future experimental findings. All lowest energy structures were found to have neutral, rather than ion-pair monomers. Near Earth's surface, we predict significant concentrations of *n* = 1–2 clusters (2.06 × 10^5^ molecules cm^–3^ and 1.13 × 10^4^, respectively); however, the absence of larger clusters indicates that TFA likely serves a negligible role in NPF.

## Graphical abstract

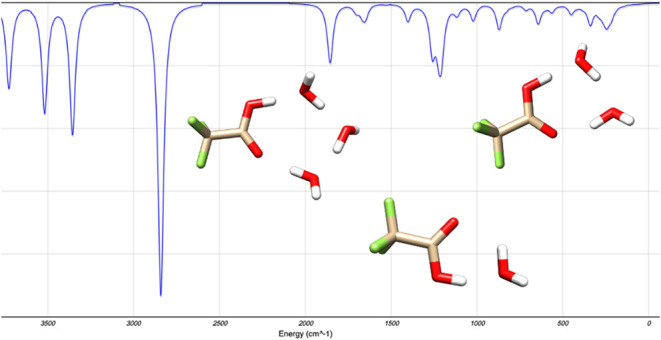

## Introduction

Cloud formation begins with cloud condensation nuclei (CCN), the atmospheric aerosols that act as nucleation sites for water droplet formation. The most recent IPCC report confirmed that CCN formation is still the largest uncertainty in climate models.^1^ Aerosols enter the atmosphere in one of two ways. They can be directly deposited via sea spray, desert dust, forest fires, factory smoke, etc., or formed in the atmosphere from gas-phase molecules, a process that is known as new particle formation (NPF). NPF can account for up to half of the CCN in the atmosphere.^2^ Thus, understanding NPF can help reduce the uncertainty in climate models.

NPF involves three steps: formation of prenucleation clusters, nucleation into clusters that are stable against evaporation, and subsequent cluster growth. The vapors driving these steps remain poorly understood. Quantum mechanical studies summarized by Elm et al. highlight the role of aerosols like sulfuric acid, water, ammonia, and amines over land, and methanesulfonic acid over oceans.^3,4^ Here, we investigate trifluoroacetic acid (TFA) and its interactions with water in the atmosphere, evaluating its thermodynamic stability and potential role in nucleation pathways.

TFA, CF_3_COOH, belongs to a class of molecules called per- and polyfluoroalkyl substances (PFAS), which are defined as molecules where the carbon chain is either completely (per-) or partially (poly-) fluorinated. PFAS are known as “forever chemicals” due to the strong C–F bonds that resist degradation. TFA is the terminal degradation product for several hydrofluorocarbons and hydrofluoroolefins, widely used as refrigerants,^5,6^ and it can also re-enter the atmosphere via sea spray.^7^ TFA has been detected in rain,^8^ dust, herbal drinks,^9^ plants,^10^ human serum,^11^ Arctic ice cores,^12,13^ and sea spray,^7^ yet all sources and pathways of TFA formation are not fully known.^14,15^ The growing accumulation of TFA has increased concern about the environmental impacts of the molecule.^5^ Recent studies suggest mammalian toxicity of TFA, including the potential for reproductive and liver damage.^16^

TFA is highly soluble in water.^17^ However, TFA is present in the atmosphere in the gas-phase,^15,18,19^ and its resilience, acidity, and ability to form hydrogen bonds suggest that it may act as a key nucleating species in NPF. Previous studies show that TFA enhances new particle formation of sulfuric acid and dimethylamine by a factor of 2.3,^20^ and methanesulfonic acid and methylamine by a factor of 7.28.^21^ Similar behavior has been reported for other fluorinated carboxylic acids: perfluoropropionic acid (PFFA), for example, forms stable hydrogen-bonded clusters with atmospheric species such as sulfuric acid, methanesulfonic acid, and monoethanolamine.^22,23^ In the present study, we consider the new particle formation of gas-phase hydrated TFA clusters.

Stable conformations of TFA-water clusters have been explored previously through both computation and spectroscopy. Maity and co-workers found that TFA-*n*H_2_O, *n* = 1–7, MP2/aug-cc-pVTZ and CCSD­(T)/aug-cc-pVDZ electronic interaction energies decrease approximately linearly with the number of water molecules until *n* = 6, at which point a sharp stabilization occurs. This additional stabilization corresponds to the formation of an ion-pair structure comprising trifluoroacetate (CF_3_COO^–^) and the hydronium ion.^24^ Numerous infrared (IR) spectroscopy studies have characterized TFA and its hydrates in the gas phase.^25–30^ Ito reported IR spectra of TFA hydrates up to TFA-*n*H_2_O, *n* = 6, isolated in argon and nitrogen matrices.^25^ Complementary C=O stretching frequencies, computed with B97–1/6–311+G**, along with earlier IR measurements,^30^ aided in the assignments of TFA-*n*H_2_O, *n* = 1–5, but not *n* = 6.^25,31,32^ As hydration increases, the conformational landscape grows rapidly, complicating the identification of low energy isomers. To manage this complexity, Ito chose to implement structural constraints of polycyclic hydrogen-bond frameworks and homodromic hydrogen-bond orientations, leading to 34 TFA-4H_2_O^31^ and 70 TFA-5H_2_O^32^ possible conformations for his assignments. Although Ito identified the vibrational signature of TFA-6H_2_O,^32^ to our knowledge, the corresponding structure remains undetermined. Features were also tentatively assigned to unidentified ion-pair species.^32^ Additional characterization using pulsed-nozzle Fourier transform microwave spectrometry has also been used to characterize clusters of TFA with 1–3 water molecules.^33^ The clusters in this study have the same conformations as those identified by infrared spectroscopy.

Those prior works provide valuable insights into the formation of TFA-water clusters and their ring-forming tendencies. However, the inability to sample the vast conformational space has limited unambiguous assignments of TFA-*n*H_2_O (*n* = 4–6) and ion-pair species.^32^ One goal of the present study is to exhaustively sample the configurational space of TFA hydrates containing up to 8 waters, validate our computational approach through comparison to microwave and infrared spectra of TFA-*n*H_2_O (*n* = 1–3), and predict the most stable isomers of TFA-*n*H_2_O (*n* = 4–8) at 0 K, including an assessment of the plausibility of ion-pair formation.

Most existing TFA-hydrate studies focus on electronic or zero-point corrected energies, limiting conclusions about cluster behavior in the troposphere. For atmospherically relevant predictions, free energies and their temperature dependence must be considered. It has been estimated that more than 8% of TFA monomers in the lower troposphere will form the TFA-H_2_O dimer, although this value likely carries substantial uncertainty due to the underestimation of binding energies.^33^ To address this, we compute relative Gibbs free energies of clusters with TFA and 1–8 waters across three temperatures spanning the troposphere: 298.15 K (near-surface), 273.15 K (midtroposphere), and 216.65 K (upper troposphere). From these free energies, we identify the most stable configurations at different regions of the troposphere, determine formation free energies, and predict the concentrations of TFA-water clusters relevant to NPF.

## Methods

The identification of structures and their energy calculations followed the computational funnel protocol in Figure 1.^3^

**Figure 1:**
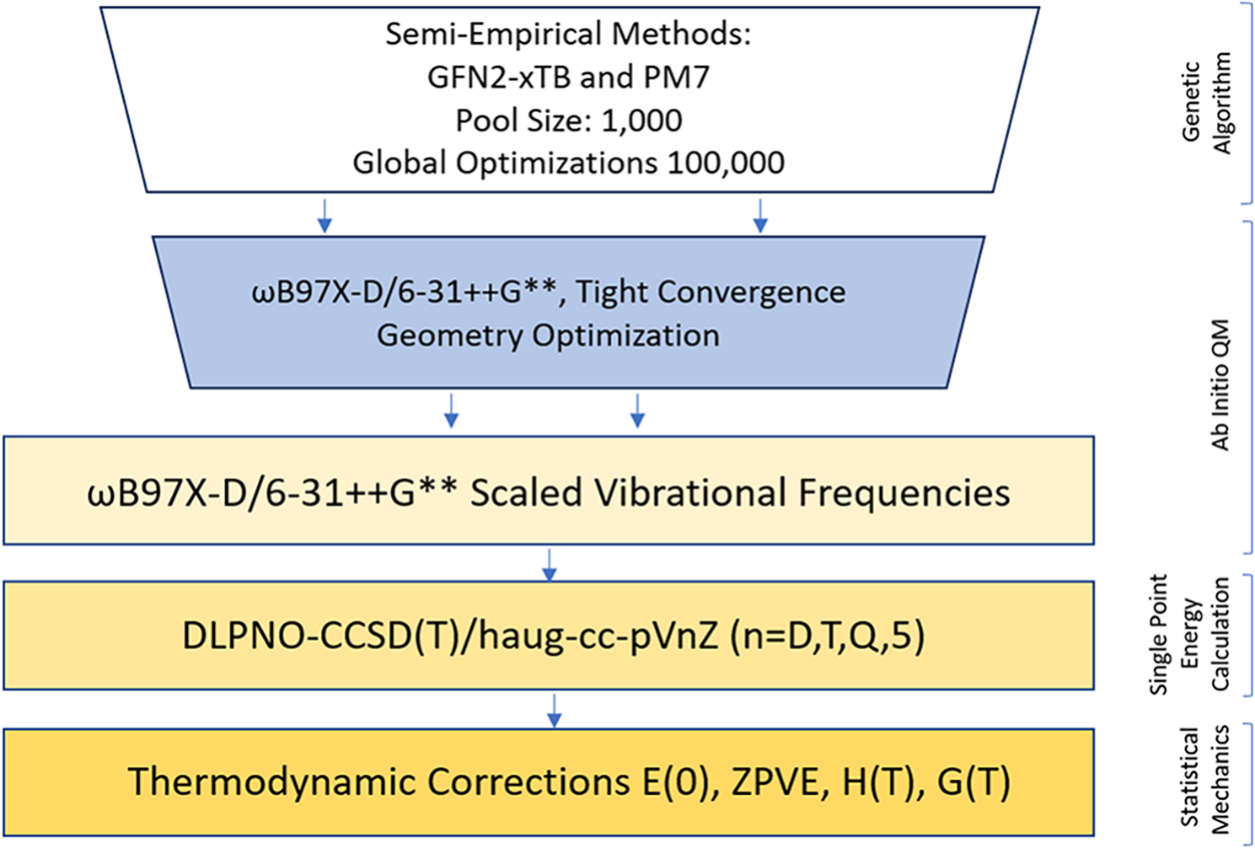
Funnel of computational methodologies used for generating molecular structures and determining the Gibbs free energies of each system.

Conformers of the TFA-*n*H_2_O (*n* = 1–8) clusters were sampled using OGOLEM,^34^ an evolutionary cluster optimization algorithm, in combination with two semiempirical methods GFN2-xTB^35,36^ and PM7.^37^ For each cluster size, we performed two independent OGOLEM searches with each semiempirical method, using a pool size of 1000 configurations per search. The resulting sets, up to ∼2000 candidate structures per cluster per method, were then combined and subjected to a similarity filter (rotational constants within 1% and energies within 0.00015 au which is 0.094 kcal/mol) to remove duplicates prior to higher-level optimization. The unique structures were then optimized with the ωB97X^38,39^ functional in Gaussian 16 Rev. B.01 ^40^ with the 6–31++G** basis set^41–43^ and Grimme’s dispersion correction^44^ (i.e., ωB97X-D), a model chemistry that has been successful in previous studies of atmospheric and water clusters.^3,45^ For all unique structures with relative electronic energies within 6 kcal/mol of the global minimum, electronic energies were refined with the domain-based local pair natural orbital approach for coupled-cluster single, double, and perturbative triples method (DLPNO–CCSD­(T))^46–49^ in Orca 6.0.0.^50^ Specifically, we used the semicanonical triples correction (T_0_). Pair natural orbital (PNO)-specific cutoffs were 10^–4^ for TCutPairs, the energetic threshold for weak and strong electron pairs; 10^–2^ for TCutDO and 10^–3^ for TCutMKN, defining the initial domains for the PNO expansion; and 3.33 × 10^–7^ for TCutPNO, the minimum occupation number for PNOs to be considered significant. This set of thresholds in ORCA defines “NormalPNO”. For the DLPNO–CCSD­(T) calculations, we used Dunning correlation-consistent basis sets with diffuse functions added to the non-hydrogen atoms, denoted haug-cc-pVNZ,^51,52^ where *N* is the zeta-level. Electronic energies for *N* = D, T, Q, 5 are provided in the Supporting Information. We found that, relative to DLPNO–CCSD­(T)/haug-cc-pV5Z, ωB97X-D/6–31++G** routinely overstabilizes the relative energies of ion-pair complexes. A correlation plot to illustrate this behavior is provided in the Supporting Information. These results can be explained by the self-interaction error in DFT,^53^ supporting the use of wave function correlation methods, like DLPNO–CCSD­(T), over density functional approximations to provide the most accurate electronic energies. Data within the main text uses electronic energies computed with DLPNO–CCSD­(T)/haug-cc-pV5Z. The number of unique structures found in the configurational sampling step, the number of unique DFT-optimized structures, and the number of DLPNO–CCSD­(T)/haug-cc-pV5Z calculations are presented in Table 1.

**Table 1:** Number of Unique Structures from the Conformational Sampling Step and the Number of Unique ωB97X-D/6-31++G** Optimized Structures with Relative DFT Electronic Energies (Δ*E*_el_) within 6 kcal/mol of the ωB97X-D/6-31++G** Minimum^a^

	sampled	ωB97X-D/6–31++G** Δ*E*_el_ < 6 kcal/mol
TFA-H_2_O	369	1
TFA-2H_2_O	1091	4
TFA-3H_2_O	1985	29
TFA-4H_2_O	3456	132
TFA-5H_2_O	4523	530
TFA-6H_2_O	5020	666
TFA-7H_2_O	5373	414
TFA-8H_2_O	5665	855

a DLPNO–CCSD­(T) single point energies were computed for the structures in the final column.

To ensure basis set convergence, we computed relative electronic energies for TFA-*n*H_2_O (*n* = 1–4) clusters with DLPNO–CCSD­(T)/haug-cc-pV6Z (also provided in the Supporting Information). Mean absolute errors for relative energies between DLPNO–CCSD­(T)/haug-cc-pV6Z and DLPNO–CCSD­(T)/haug-cc-pV5Z are 0.019, 0.013, 0.015, and 0.017 kcal/mol for clusters with 1,2,3, and 4 waters, respectively. We conclude that haug-cc-pV5Z basis sets are sufficient for computing accurate Gibbs free energies of hydrated TFA clusters. Mean absolute errors between pairs of basis sets with lower-zeta levels are in the Supporting Information.

DLPNO–CCSD­(T) energies can be sensitive to the PNO thresholds, especially for noncovalent systems.^47^ To assess these effects for the TFA-5H_2_O clusters, we benchmarked ORCA’s TightPNO settings (TCutPairs = 10^–5^, TCutDO = 5 × 10^–3^, TCutMKN = 10^–3^, and TCutPNO = 10^–7^) and NormalPNO settings against canonical CCSD­(T) with the haug-cc-pVDZ basis sets for six structures (four neutral, two ion-pair). TightPNO DLPNO–CCSD­(T) reproduces canonical CCSD­(T) relative energies to within 0.1 kcal/mol for the neutral clusters and 0.3 kcal/mol for the ion-pair clusters. NormalPNO remains reasonably accurate, reproducing canonical CCSD­(T) relative energies within 0.3 kcal/mol for the neutral clusters and 0.7 kcal/mol for the ion-pair clusters. These differences lead to only minor changes in the rank ordering of the six benchmark structures.

We also compared NormalPNO and TightPNO directly using haug-cc-pVQZ basis sets for a larger group of (467) TFA-5H_2_O clusters. NormalPNO systematically increases the relative energies of ion-pair structures (mean = +0.318 kcal/mol) but slightly decreases those of the neutral clusters (mean = −0.058 kcal/mol.). Thus, ion-pair systems are more sensitive to PNO truncation than neutral clusters.

This distinction matters because the PNO thresholds introduce different systematic errors for neutral and ion-pair clusters; without recognizing this, one might misinterpret energy trends or relative stabilities across chemically distinct classes of structures. However, the ion-pair structures all have high relative energies, so the effect of including tighter PNO cutoffs will not significantly change the relative energy rankings of the lowest energy structures. Furthermore, the small changes in relative energies for neutral clusters do not justify the increased computational effort of TightPNO thresholds. Therefore, all DLPNO energies reported in the main text were computed with NormalPNO settings.

Enthalpies at 0 K and Gibbs free energies at *T* > 0 K were obtained by computing the remaining enthalpy and/or entropy terms at a standard state of 1 atm pressure with the thermo.pl script from the National Institute of Standards and Technology,^54^ with ωB97X-D/6–31++G** frequencies scaled by 0.971 ^55^ to approximate anharmonicity. The most common method for calculating anharmonic frequencies is second-order vibrational perturbation theory (VPT2).^56,57^ Like any perturbation theory, this method can suffer from numerical errors. Alternatively, scaling harmonic frequencies by a factor specific to the level of theory is a widely used method for accounting for anharmonicity. For atmospheric clusters, we have found this technique to be an effective method for computing Gibbs free energies, even for systems with many low-frequency modes where VPT2 would be numerically unstable.^58–60^

The Boltzmann-weighted population (BP) of each isomer is calculated according to

$$\mathrm{BP}=\frac{e^{-\Delta {G{}^{\circ}}_{i}/\mathrm{RT}}}{\sum_{i}e^{-\Delta {G{}^{\circ}}_{i}/\mathrm{RT}}}$$

where Δ*G*°*_i_* is the Gibbs free energy of isomer *i* relative to the global minimum, *R* is the gas constant, and *T* is temperature. Boltzmann populations are reported in the Supporting Information alongside unscaled rotational constants and principal axis dipole moments.

Binding free energies, Δ*G*°_binding_, are calculated as

$${\Delta G^{{}^{\circ}}}_{\mathrm{binding}}={G^{{}^{\circ}}}_{\mathrm{TFA}‐nH_{2}O}-{G^{{}^{\circ}}}_{\mathrm{TFA}}-{{\mathrm{nG}}^{{}^{\circ}}}_{H_{2}O}$$

where *G*°_TFA‑*n*H_2_O_ is the Gibbs free energy of the lowest-energy cluster of TFA-*n*H_2_O, *G*°_TFA_ is the Gibbs free energy of TFA, and *nG*°_H_2_O_ is n times the Gibbs free energy of water. At *T* > 0 K, the Gibbs free energies used in this equation account for the presence of multiple conformers, {*A*}, that are thermodynamically accessible through the multiconformer correction^61^

$${\Delta G^{{}^{\circ}}}_{\left\{A\right\}}=-\mathrm{RT}\;\ln(\sum_{i\in \left\{A\right\}}e^{-\Delta {G{}^{\circ}}_{i}/\mathrm{RT}})$$

where Δ*G°*_*i*_ is the Gibbs free energy of conformer *i* relative to the minimum energy conformer. Estimated equilibrium concentrations of each TFA-*n*H_2_O cluster at 216.65 and 298.15 K were calculated from the binding Gibbs free energies and estimated initial concentrations of TFA and water. The initial water concentrations were 7.7 × 10^17^ cm^–3^ at 298.15 K and 9.9 × 10^14^ cm^–3^ at 216.65 K. These correspond to 100% humidity at the bottom and top of the troposphere.^62^ The initial concentration of TFA at 298.15 K was set to 5.0 × 10^6^ cm^–3^, based on previous measurements in Beijing^18,19^ and Shanghai.^20^ At 216.65 K, we decreased the TFA concentration by 3 orders of magnitude to account for atmospheric thinning at the top of the troposphere. This is an estimate, used in previous studies,^63^ based on the decrease in the concentration of water.

## Results and Discussion

### TFA Hydrates at 0 K

The minimum energy structures of TFA-*n*H_2_O at 0 K, shown in Figure 2, are exclusively neutral monomers. The waters form ring-like hydrogen-bonding motifs with the COOH group, and these rings increase in dimensionality as the number of waters increases. No hydrogen bonding to fluorine atoms is observed; it has previously been reported that fluorine’s high electronegativity and low polarizability cause it to be a poorer hydrogen bond acceptor than oxygen.^64^ From TFA-H_2_O to TFA-4H_2_O, the acidic hydrogen in TFA progressively shifts away from its bonded oxygen and toward the oxygen of its hydrogen-bonded water. However, at TFA-5H_2_O, this trend is no longer observed, likely due to the increasingly robust hydrogen bond network. The binding enthalpies of these clusters range from −8.60 to −61.66 kcal/mol (1 water to 8 waters, respectively) and are listed in the Supporting Information.

**Figure 2:**
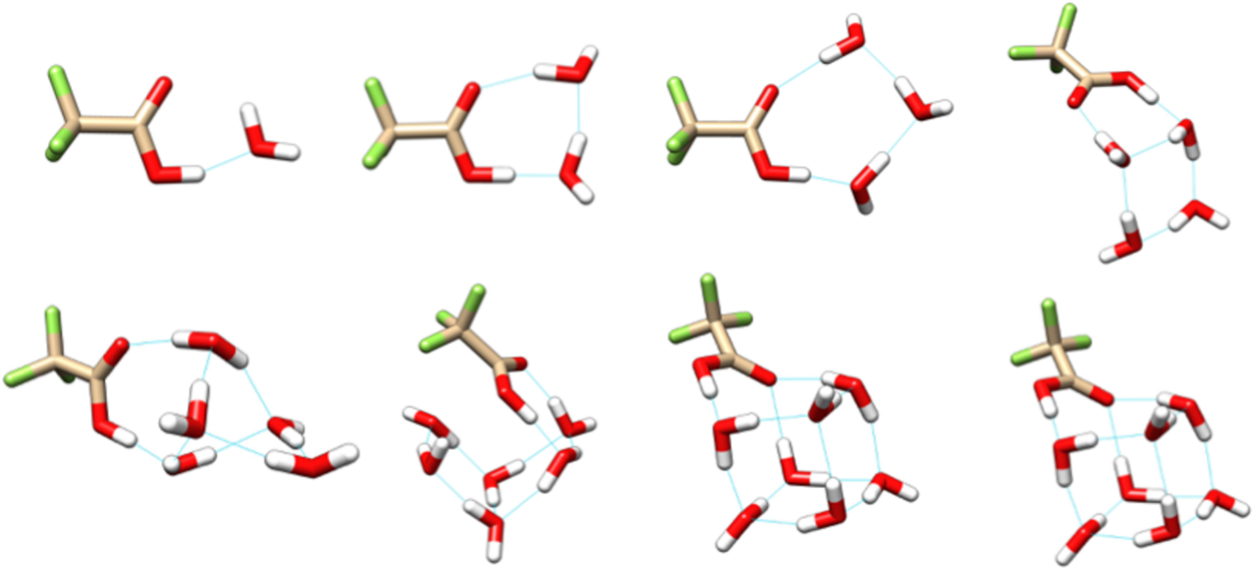
Minimum energy structures according to DLPNO–CCSD­(T)/haug-cc-pV5Z//ωB97X-D/6–31++G** enthalpies at 0 K. Anharmonicity is approximated by scaling the harmonic ωB97X-D/6–31++G** frequencies by 0.971.

Our computational funnel yields minimum energy TFA-H_2_O, TFA-2H_2_O, and TFA-3H_2_O structures that agree with existing microwave and vibrational spectra. The stretching frequency of the C=O bond is often used to determine the number of waters in each observed structure, whereas rotational constants are used to differentiate among specific isomers. Therefore, computed and experimental C=O frequencies (scaled by 0.971) and rotational constants (unscaled) are compared in Table S2.

Previously published computational values in Table S2 include MP2/6–311++G** rotational constants (with and without the counterpoise correction) and B971/6–311++G** vibrational frequencies. In general, the ωB97X-D/6–31++G** rotational constants agree with experiment well, and the agreement is better than the MP2 results without the counterpoise correction. Furthermore, ωB97X-D/6–31++G** computes the vibrational frequency of the C=O stretching more accurately than B971/6–311++G**. Our DFT methodology uses a double-ζ basis set only, and no counterpoise correction, making the calculations more computationally feasible, especially for larger systems. Due to its agreement with experiment and MP2 theory, ωB97X-D/6–31++G** is a computationally efficient and appropriate method for optimizing geometries and computing frequencies of TFA and its hydrates.

Experimental identification of TFA-4H_2_O and TFA-5H_2_O clusters has been attempted previously^31,32^ but has been hindered by the lack of microwave spectroscopy results and, before the present study, an incomplete exploration of the potential energy surfaces. The isomer sets examined in those studies do not include all low-lying minima. Our sampling protocol predicts nine TFA-4H_2_O and 34 TFA-5H_2_O isomers within 1 kcal/mol of the DLPNO–CCSD­(T)/haug-cc-pV5Z//ωB97X-D/6–31++G** minima at 0 K. Representative structures that illustrate the dominant bonding motifs are shown in Figure 3, and images of all structures are provided in the Supporting Information.

**Figure 3:**
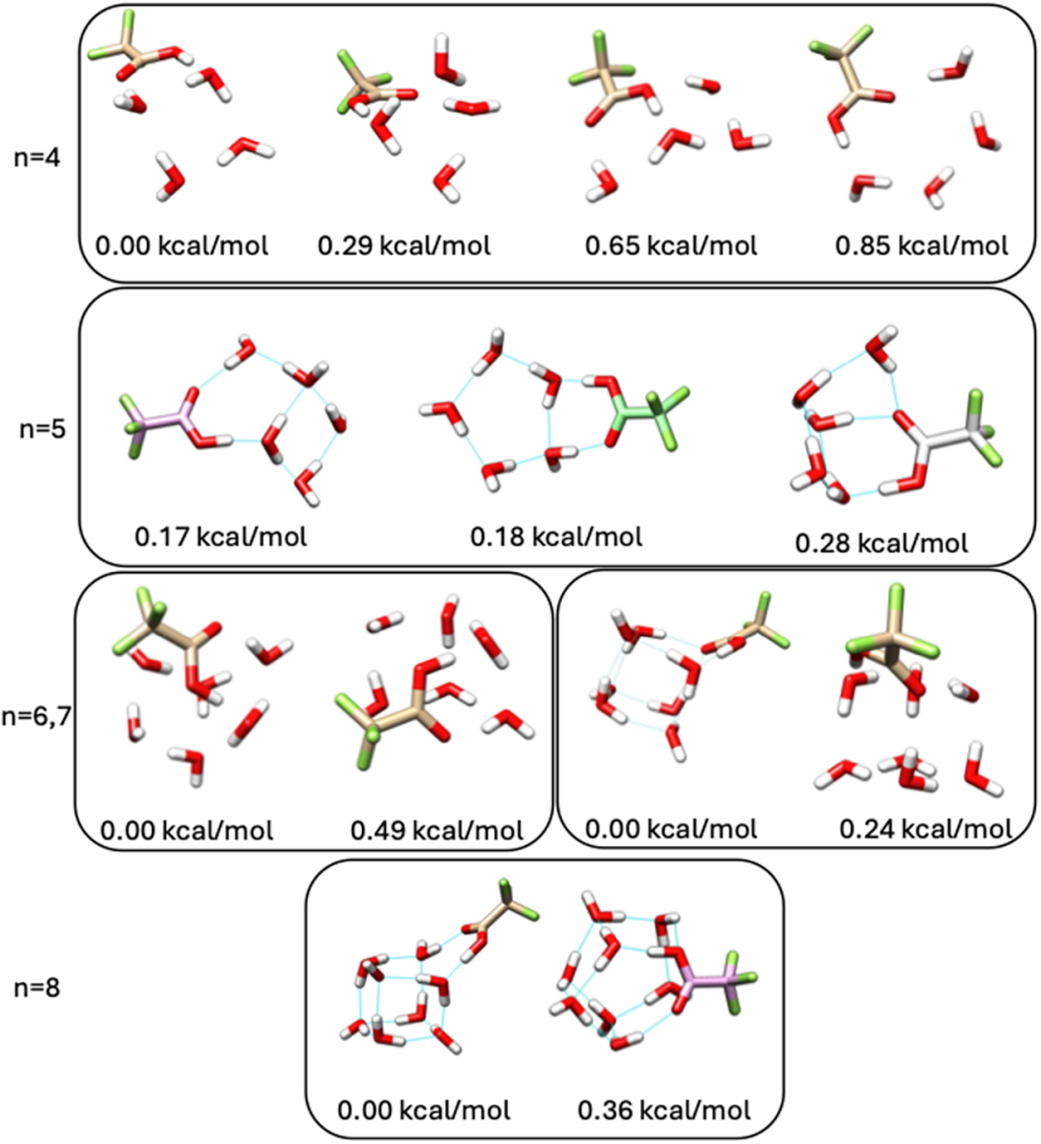
Representative low energy structures of TFA-*n*H_2_O with *n* = 4–6 at 0 K, illustrating the dominant bonding motifs within the full configurational set. DLPNO–CCSD­(T)/haug-cc-pV5Z//ωB97X-D/6–31++G** relative enthalpies are reported below each structure in kcal/mol.

The nine TFA-4H_2_O isomers within 1 kcal/mol of the minimum at 0 K can be divided into four groups, displayed in Figure 3, where naming has already been established in ref 31 (isomers of Figure 4’s ‘[2 + 2]­2d’, ‘[4 + 0]’, and ‘[2 + 2]­3d’; and Figure 1 ’s structure e ‘[2 + 1+1]­(2d,2u)’ in ref 31). We provide the names here solely to facilitate comparison between our results and those previously reported. We note that the ‘[2 + 1+1]­(2d,2u)’ structure in ref 31 was 6.5 kcal/mol higher in energy than the minimum; however, we identify isomers of this structure with relative energies of 0.29 and 0.38 kcal/mol. The 34 TFA-5H_2_O isomers generally contain two or three waters forming a ring with TFA, with the remaining waters forming an additional ring, either exclusively with water molecules or bridging between waters and TFA. The many possibilities for this arrangement lead to the 34 TFA-5H_2_O isomers. Table 2 shows that our C=O stretching frequencies agree with experiment; however, microwave spectroscopy studies are needed to confirm the presence and conformations of the many low-lying isomers of TFA-4H_2_O and TFA-5H_2_O at 0 K. (For relevance to jet-cooled experimental conditions, relative energetics of low-lying isomers at 2 K are provided in the Supporting Information.)

**Figure 4:**
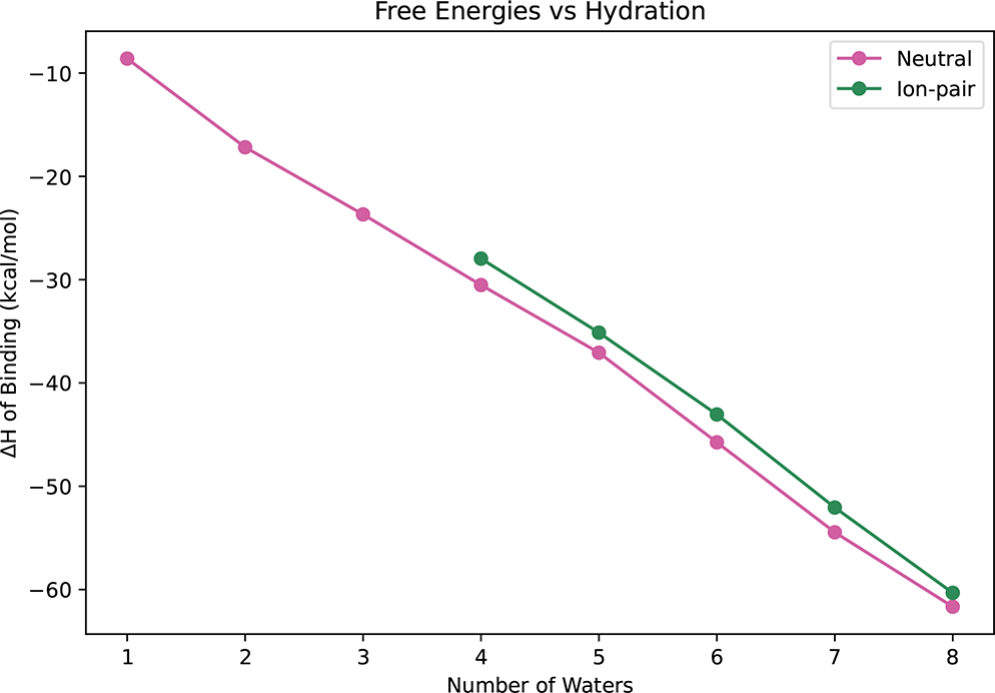
Binding enthalpies for the lowest-energy neutral TFA-*n*H_2_O, *n* = 1–8, clusters and the lowest-energy ion-pair TFA-*n*H_2_O, *n* = 4–8, clusters at 0 K. Binding enthalpies are computed with DLPNO–CCSD­(T)/haug-cc-pV5Z//ωB97X-D/6–31++G**. Ion-pair enthalpies are higher than their neutral counterparts by 2.56 kcal/mol for TFA-4H_2_O, 1.94 kcal/mol for TFA-5H_2_O, 2.70 kcal/mol for TFA-6H_2_O, 1.85 kcal/mol for TFA-7H_2_O, and 1.34 kcal/mol for TFA-8H_2_O.

**Table 2:** Theoretical (ωB97X-D/6-31++G**) and Experimental^25,32^ Results for the Lowest Energy Isomers of TFA-*n*H_2_O, *n* = 4-8, at 0 K: Rotational Constants (A, B, C) in MHz and C=O Stretching Frequencies [v­(C=O)] in cm^–1^ Scaled by 0.971

	TFA-4H_2_O	TFA-5H_2_O	TFA-6H_2_O	TFA-7H_2_O	TFA-8H_2_O
A Calc	1380	1448	773	734	687
B Calc	368	262	412	243	170
C Calc	345	259	362	237	164
v(C=O) Calc	1770	1794	1789	1752	1835
v(C=O) Expt	1723^a^, 1765^b^	1728^b^	1737^b^		

a Value from ref 32.

b Value from ref 25.

The measured C=O stretching frequency of TFA-6H_2_O is reasonable according to our ωB97X-D calculations (Table 2). We identify four isomers of TFA-6H_2_O that lie within 1 kcal/mol of the minimum energy structure at 0 K. These four isomers have scaled C=O stretching frequencies between 1767 and 1794 cm^–1^, consistent with the experimental value of 1737 cm^–1^. The lowest energy isomer has a C=O stretching frequency of 1789 cm^–1^. Interestingly, the low-lying TFA-6H_2_O isomers are structurally very similar to the book and prism pure water hexamers^65^ but exhibit minor rearrangements in order to accommodate hydrogen bonding to TFA. See Figure 3.

Two TFA-7H_2_O structures lie within 1 kcal/mol of the minimum at 0 K. These two are cube-like structures, and the second-lowest energy structure resembles the water heptamer prism with an attached TFA molecule.^66^ Among the 20 TFA-8H_2_O structures, many feature square or pentagonal faces, with TFA hydrogen bonding along the cluster edge. Across all TFA-*n*H_2_O (*n* = 1–8) clusters, every isomer within 1 kcal/mol includes hydrogen bonding to the COOH group, while the C–F groups never participate. Rotational constants for TFA-*n*H_2_O clusters with 4 to 8 waters are provided in Table 2. To our knowledge, these clusters have not been identified through microwave spectroscopy. The Supporting Information provides all low-lying minima for TFA-*n*H_2_O clusters, their rotational constants, principal axis dipole moments, and vibrational frequencies.

At 0 K, all of the lowest-energy structures consist of neutral monomers. The lowest relative energies at 0 K of ion-pair clusters are 2.56 kcal/mol for TFA-4H_2_O, 1.94 kcal/mol for TFA-5H_2_O, 2.70 kcal/mol for TFA-6H_2_O, 1.85 kcal/mol for TFA-7H_2_O, and 1.34 kcal/mol for TFA-8H_2_O. The actual values are expected to be ∼0.3 kcal/mol lower than those reported, due to the use of NormalPNO cutoffs as discussed in the methodology section. Each of these structures have an overall principal axis dipole moment above 3.8 D, suggesting that these complexes, especially TFA-8H_2_O, may be detectable via high-resolution rotational spectroscopy.

Gibbs free energies are negative for the cluster formation of both the neutral and ion-pair minima. The binding enthalpies are plotted in Figure 4 and listed in Tables S3 and S4. For the neutral clusters, binding enthalpies range from −8.60 to −61.66 kcal/mol as hydration increases from 1 to 8 waters. The enthalpies follow a near-linear trend, which agrees with previous reports.^24^ Our computed enthalpies are smaller in magnitude (less negative) than those previously obtained with MP2/aug-cc-pVTZ and CCSD­(T)/aug-cc-pVDZ,^24^ primarily because the DLPNO–CCSD­(T)/haug-cc-pV5Z//ωB97X-D/6–31++G** methodology more accurately captures intermolecular interactions and reduces basis set superposition error. For *n* = 4–7, geometric differences between studies also contribute, as the structures identified here are lower in energy according to ωB97X-D/6–31++G** and DLPNO–CCSD­(T)/haug-cc-pV5Z.

The linear trend remains for the binding enthalpies for the lowest-energy ion-pair clusters (*n* = 4–8). For each cluster, the ion-pair enthalpies of binding are smaller in magnitude (less negative) than their neutral counterparts, reflecting the reduced stability of the ion-pair structures. No ion-pair clusters with ωB97X-D/6–31++G** relative energies within 6 kcal/mol were found for clusters containing one to three waters.

### TFA Hydrates at Tropospheric Temperatures

At higher temperatures, Gibbs free energies change, which often leads to new lowest-free-energy structures relative to the 0 K minima. Clusters that maintain the same lowest energy structure at 0, 216.65, 273.15, and 298.15 K are TFA-H_2_O, TFA-7H_2_O, and TFA-8H_2_O. All other clusters adopt different minima at these higher temperatures, shown in Figure 5. These higher-temperature structures frequently feature additional dangling hydrogens (those not participating in a hydrogen bond) relative to their 0 K low-energy structures. This causes an increase in entropy which favors a lower Gibbs free energy. For example, the low energy structures TFA-4H_2_O at 0 K (Figure 2) contains three dangling hydrogens, whereas the two low energy structures at higher temperatures (Figure 5) each contain four.

**Figure 5:**
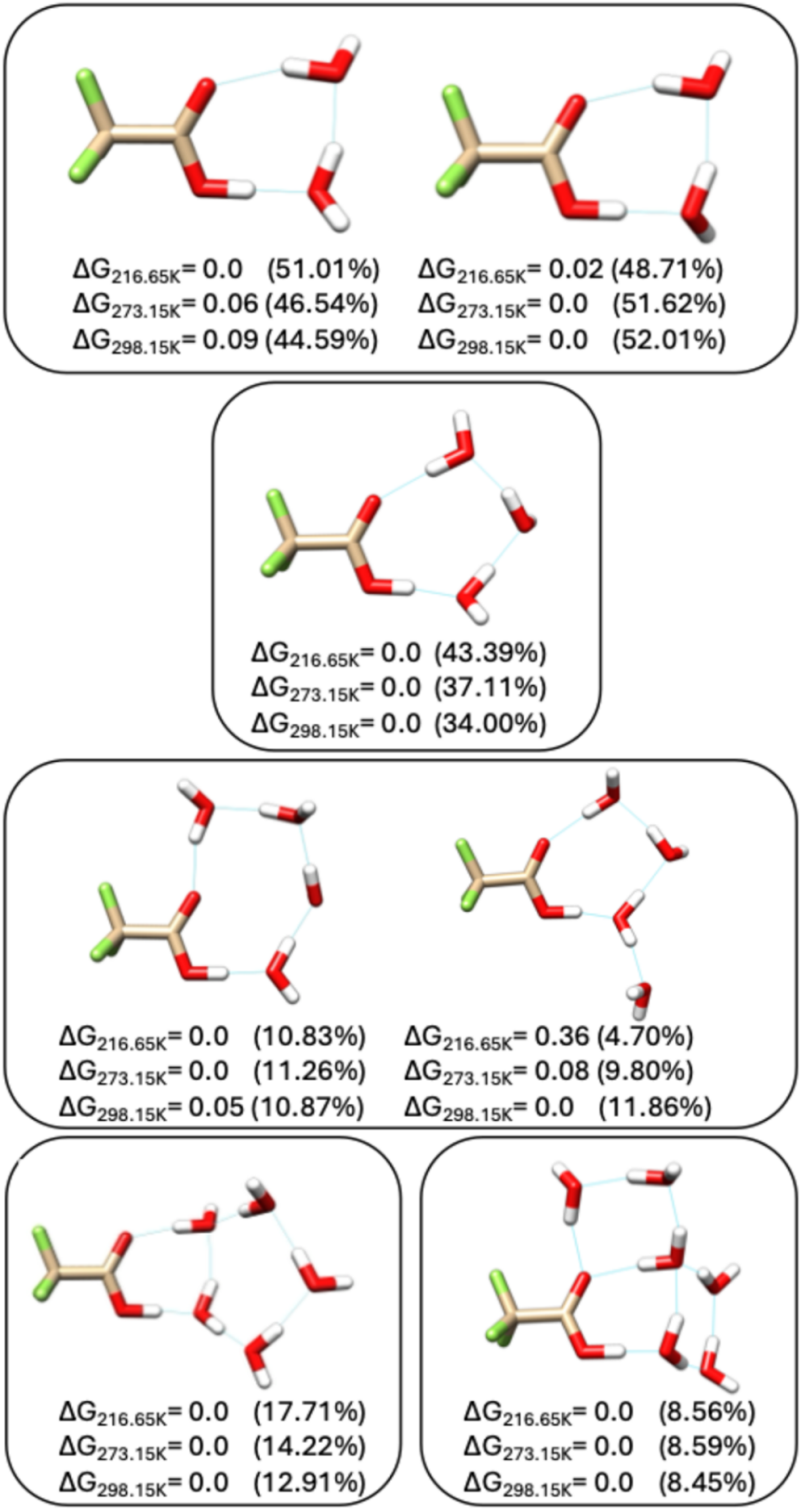
Minimum energy structures of TFA-*n*H_2_O with *n* = 2–6 at 216.65, 273.15, and 298.15 K. DLPNO–CCSD­(T)/haug-cc-pV5Z//ωB97X-D/6–31++G** relative Gibbs free energies are reported below each structure in kcal/mol. Boltzmann populations are given in parentheses. Clusters not shown retain the same lowest-energy structures at 0 K.

The increasing importance of entropy as temperature increases leads to multiple low-lying isomers. Predicted Boltzmann populations provide a measure of isomer abundances at a given temperature. At 298.15 K, there are 1 TFA-H_2_O, 3 TFA-2H_2_O, 14 TFA-3H_2_O, 35 TFA-4H_2_O, 44 TFA-5H_2_O, 47 TFA-6H_2_O, 32 TFA-7H_2_O, and 49 TFA-8H_2_O isomers with Boltzmann populations above 0.5%. Of these, one structure is an ion-pair complex. It contains eight waters, has a Boltzmann population of 0.61%, and a relative Gibbs free energy of binding at 298.15 K of 1.73 kcal/mol. Boltzmann populations for all isomers are available in the Supporting Information.

At 216.65 K, the Gibbs free energies of binding (relative to infinitely separated monomers) are negative for all clusters, ranging from −2.64 to −12.06 kcal/mol (shown in Figure 6). Gibbs free energies of binding are moderate at 273.15 K: ranging from −0.94 to 2.34 kcal/mol as hydration increases. The energies approach 8.69 kcal/mol at 298.15 K. At the two higher temperatures, Gibbs free energies generally increase with each additional water.

**Figure 6:**
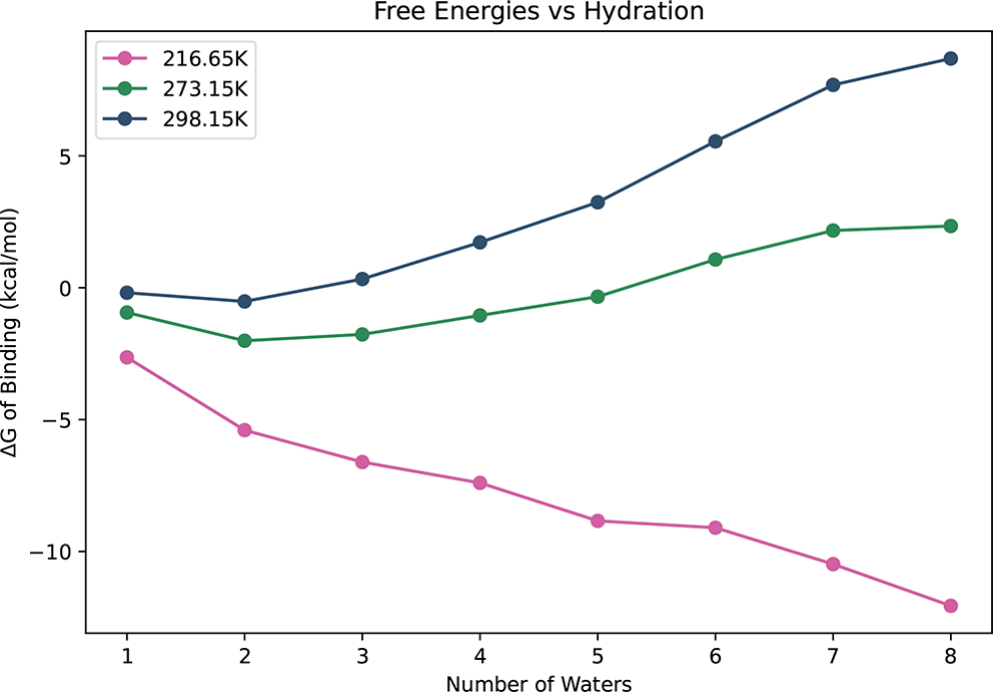
Binding Gibbs free energies for TFA-*n*H_2_O, *n* = 1–8, at 216.65, 273.15, and 298.15 K. Gibbs free energies are computed with DLPNO–CCSD­(T)/haug-cc-pV5Z//ωB97X-D/6–31++G**.

Table 3 reports the non-negligible concentrations of clusters in the atmosphere, whereas concentrations of all TFA-*n*H_2_O (*n* = 1–8) clusters are provided in the Supporting Information. At the top of the troposphere (216.65 K), there are small concentrations of TFA-H_2_O (66.5 clusters cm^–3^) and TFA-2H_2_O (1.18 clusters cm^–3^). At surface-level (298.15 K), there are notable amounts of TFA-*n*H_2_O (*n* = 1–3): 2.06 × 10^5^ clusters cm^–3^ for one water, 1.13 × 10^4^ for 2 waters, and 84 for three waters. Based on these predictions, we expect that 4.4% of atmospheric TFA will form hydrates at 298.15 K, with 4% of the TFA monomers forming the TFA-H_2_O dimer. For comparison, the predicted water hexamer concentration at 298.15 K is 3 × 10^4^ clusters cm^–3^,^67^ which is similar to predicted concentrations of TFA-2H_2_O and lower than predicted concentrations of the TFA-H_2_O dimer. For both temperatures, hydrates with *n* > 3 have concentrations below 1 cluster cm^–3^, indicating that these small clusters are unlikely to act as significant nuclei in the early stages of particle formation.

**Table 3:** Equilibrium Concentrations of Clusters at 216.65 and 298.15 K^a^

	216.65 K	298.15 K
TFA	4.93 × 10^3^	4.78 × 10^6^
H_2_O	9.90 × 10^14^	7.7 × 10^17^
TFA-H_2_O	66.4	2.06 × 10^5^
TFA-2H_2_O	1.18	1.13 × 10^4^
TFA-3H_2_O	5.74 × 10^–4^	84.0

a Initial concentrations of the monomers at 216.65 K were TFA = 5.0 × 10^3^ molecules cm^–3^ and H_2_O = 9.9 × 10^14^ molecules cm^–3^. Initial concentrations of the monomers at 298.15 K were TFA = 5.0 × 10^6^ molecules cm^–3^ and H_2_O = 7.7 × 10^17^ molecules cm^–3^.

## Conclusions

We have investigated the stability of gas-phase TFA, the simplest “forever chemical”, microsolvated with one to eight water molecules at 0 K and at representative temperatures of the troposphere, ranging from 216.65 to 298.15 K. Using extensive configurational sampling and highly accurate computational methods, we identified low-energy structures, computed Gibbs free energies, and predicted atmospheric concentrations for each cluster.

The rotational constants of the lowest energy TFA-*n*H_2_O structures for *n* = 1–3 agree well with existing microwave spectroscopic measurements, supporting the reliability of our approach. For clusters with *n* = 4–8, we report rotational constants and C=O stretching frequencies for the predicted low-energy structures. All possess nonzero overall dipole moments. Notably, TFA-*n*H_2_O with *n* = 4, 6, and 7 have fewer than ten isomers within 1 kcal/mol of their minima, whereas experimental identification of other larger clusters will be more challenging: we predict 34 isomers of TFA-5H_2_O and 20 isomers of TFA-8H_2_O within this energetic window. All 0 K minima are composed of neutral monomers; ion-pair structures only become competitive at *n* = 4, but even then, they remain 1.3–3 kcal/mol higher in energy.

At tropospheric temperatures, entropic effects reorder the relative stabilities of the isomers. At 298.15 K, we find 1 TFA-H_2_O, 3 TFA-2H_2_O, 14 TFA-3H_2_O, 35 TFA-4H_2_O, 44 TFA-5H_2_O, 47 TFA-6H_2_O, 32 TFA-7H_2_O, and 49 TFA-8H_2_O structures with Boltzmann populations above 0.5%. Although cluster formation is thermodynamically favorable at 216.65 K, it becomes increasingly unfavorable with rising temperature. Concentrations of TFA hydrates will be negligible at the top of the troposphere. Near the surface, however, we predict concentrations of 2.06 × 10^5^ molecules cm^–3^ for TFA-H_2_O and 1.13 × 10^4^ for 2TFA-H_2_O, with progressively lower concentrations for larger clusters.

Overall, while the microsolvation of TFA is thermodynamically accessible under cold conditions, the decreasing stability and abundance of larger clusters suggest that pure TFA-*n*H_2_O species are unlikely to grow into particles relevant for atmospheric nucleation.

## Supplementary Material





## Notes

Spreadsheets of electronic energies, Gibbs free energies, and Boltzmann populations for all clusters and TFA and water monomers. XYZ files and harmonic frequencies for all low-lying structures (ZIP)
Analysis of NormalPNO and TightPNO cutoffs, a comparison of ωB97X-D and DLPNO–CCSD­(T) relative energies, evaluation of basis set convergence with DLPNO–CCSD­(T)/haug-cc-pVNZ, experimental and theoretical spectroscopic results, Gibbs free energies of binding, and predicted concentrations of all clusters (PDF)
