# Supplementary material for: The Hydration of Trifluoroacetic Acid from 0 to 298 K
Source: J Phys Chem A. 2026 Jan 20;130(4):927–35. doi: 10.1021/acs.jpca.5c08151 (PMC12862820; doi:10.1021/acs.jpca.5c08151)
Supplement: Supplementary file 1 [file jp5c08151_si_001.zip › TFA_SI/Figures High Temps.pptx]

## Slide 1
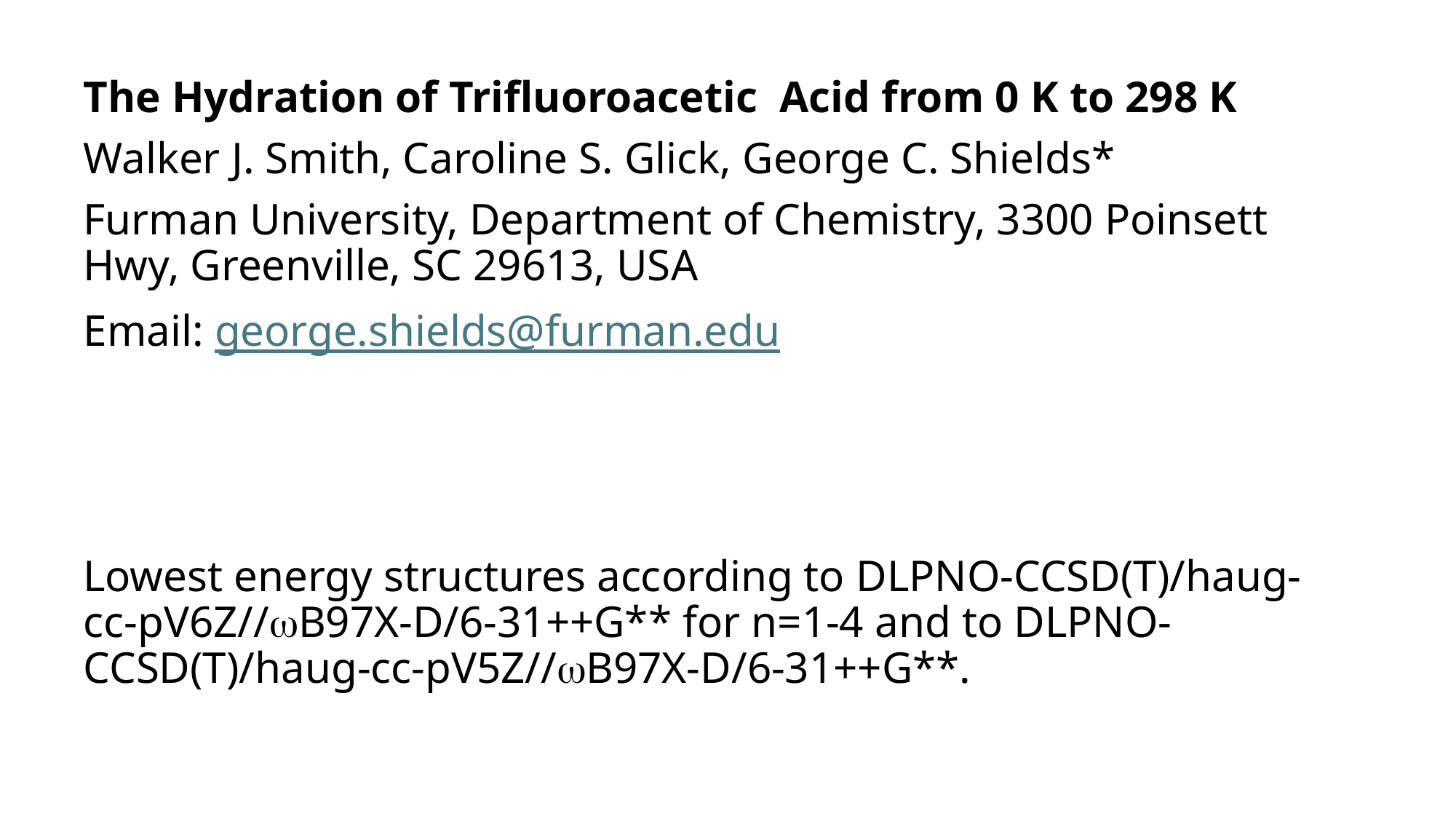

The Hydration of Trifluoroacetic  Acid from 0 K to 298 K
Walker J. Smith, Caroline S. Glick, George C. Shields*
Furman University, Department of Chemistry, 3300 Poinsett Hwy, Greenville, SC 29613, USA
Email: george.shields@furman.edu
Lowest energy structures according to DLPNO-CCSD(T)/haug-cc-pV6Z//ωB97X-D/6-31++G** for n=1-4 and to DLPNO-CCSD(T)/haug-cc-pV5Z//ωB97X-D/6-31++G**.

## Slide 2
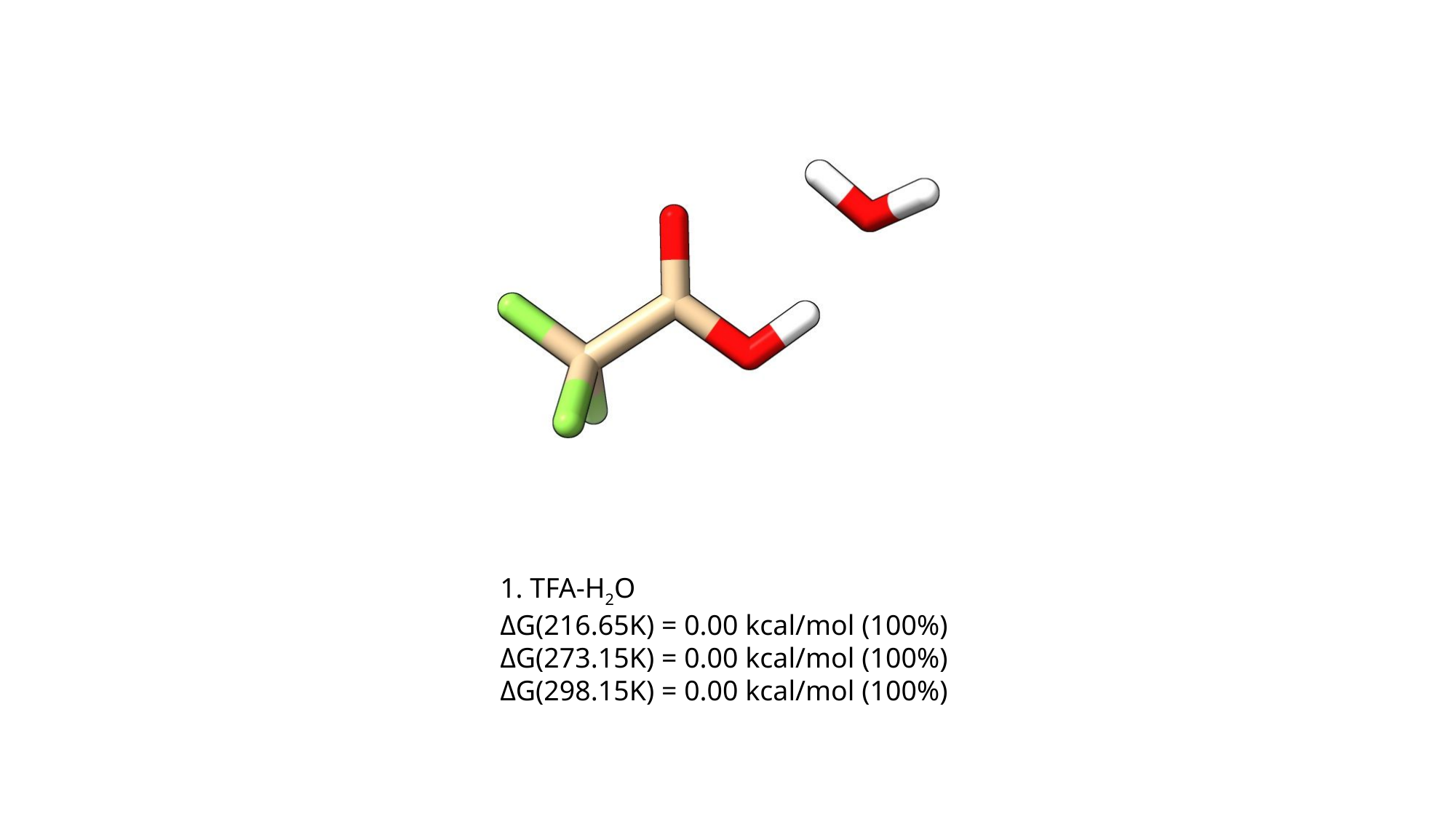

1. TFA-H2O
ΔG(216.65K) = 0.00 kcal/mol (100%)
ΔG(273.15K) = 0.00 kcal/mol (100%)
ΔG(298.15K) = 0.00 kcal/mol (100%)

## Slide 3
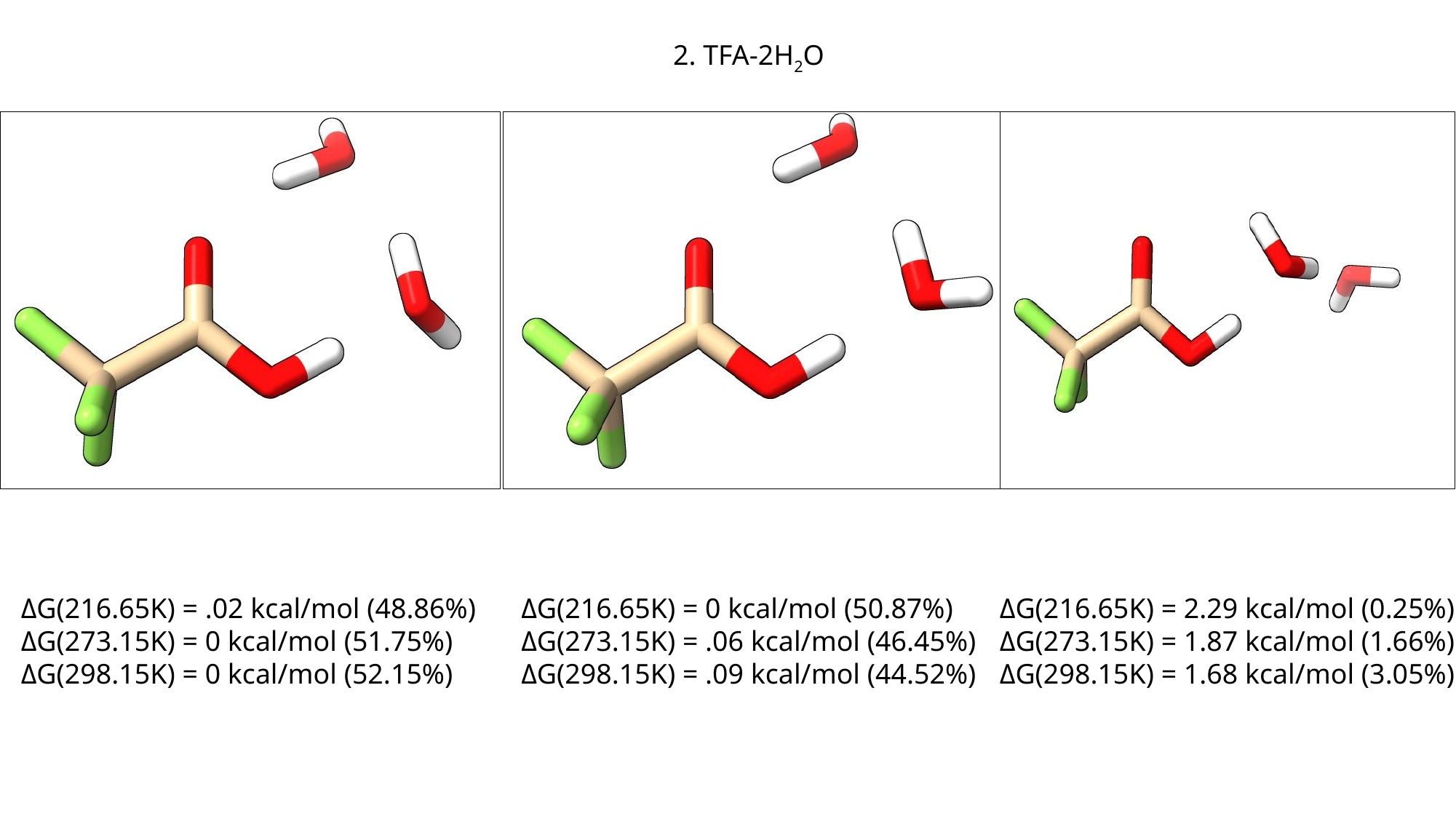

2. TFA-2H2O
ΔG(216.65K) = .02 kcal/mol (48.86%)
ΔG(273.15K) = 0 kcal/mol (51.75%)
ΔG(298.15K) = 0 kcal/mol (52.15%)
ΔG(216.65K) = 0 kcal/mol (50.87%)
ΔG(273.15K) = .06 kcal/mol (46.45%)
ΔG(298.15K) = .09 kcal/mol (44.52%)
ΔG(216.65K) = 2.29 kcal/mol (0.25%)
ΔG(273.15K) = 1.87 kcal/mol (1.66%)
ΔG(298.15K) = 1.68 kcal/mol (3.05%)

## Slide 4
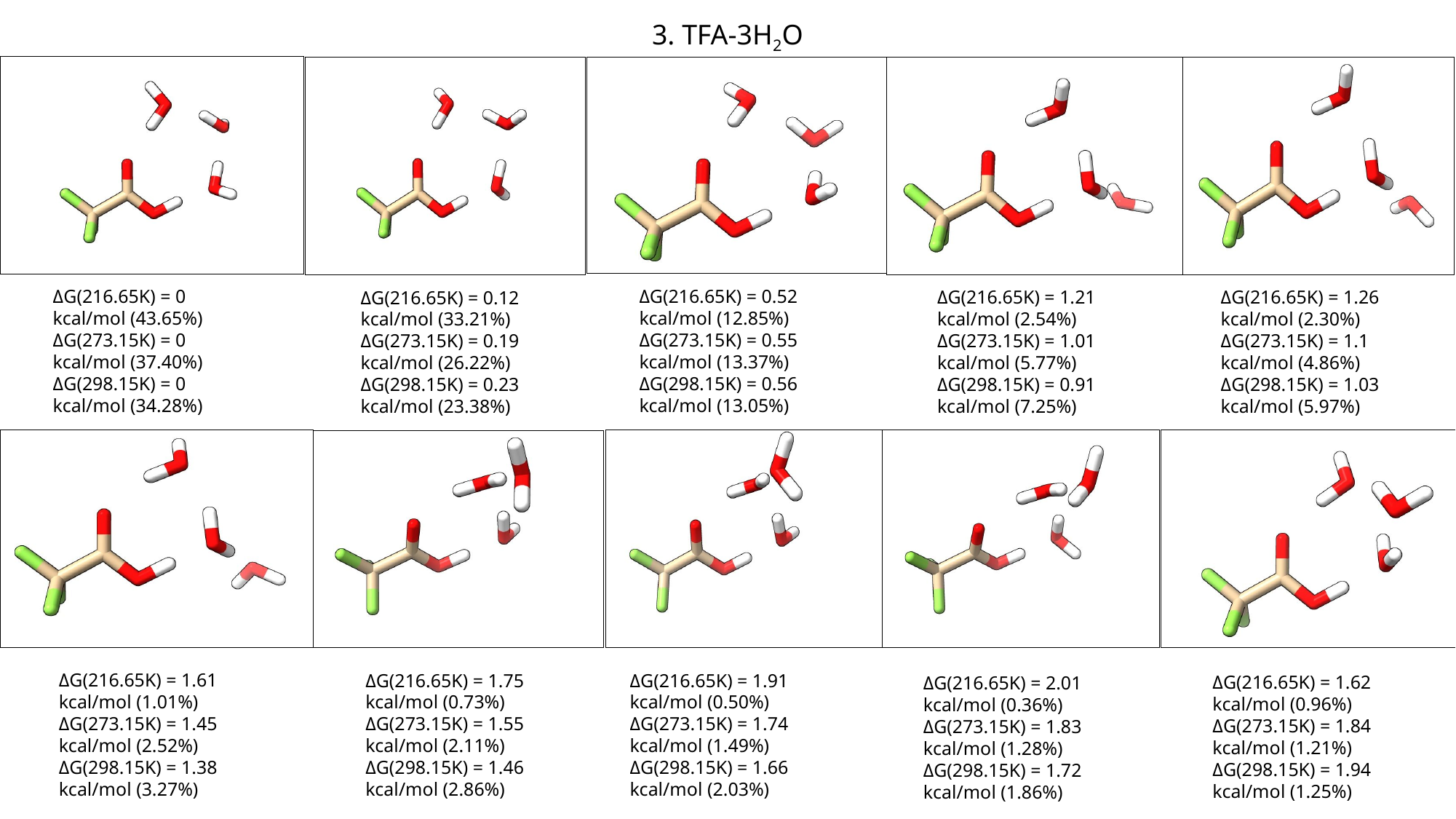

3. TFA-3H2O
ΔG(216.65K) = 0 kcal/mol (43.65%)
ΔG(273.15K) = 0 kcal/mol (37.40%)
ΔG(298.15K) = 0 kcal/mol (34.28%)
ΔG(216.65K) = 0.52 kcal/mol (12.85%)
ΔG(273.15K) = 0.55 kcal/mol (13.37%)
ΔG(298.15K) = 0.56 kcal/mol (13.05%)
ΔG(216.65K) = 1.21 kcal/mol (2.54%)
ΔG(273.15K) = 1.01 kcal/mol (5.77%)
ΔG(298.15K) = 0.91 kcal/mol (7.25%)
ΔG(216.65K) = 1.26 kcal/mol (2.30%)
ΔG(273.15K) = 1.1 kcal/mol (4.86%)
ΔG(298.15K) = 1.03 kcal/mol (5.97%)
ΔG(216.65K) = 0.12 kcal/mol (33.21%)
ΔG(273.15K) = 0.19 kcal/mol (26.22%)
ΔG(298.15K) = 0.23 kcal/mol (23.38%)
ΔG(216.65K) = 1.61 kcal/mol (1.01%)
ΔG(273.15K) = 1.45 kcal/mol (2.52%)
ΔG(298.15K) = 1.38 kcal/mol (3.27%)
ΔG(216.65K) = 1.75 kcal/mol (0.73%)
ΔG(273.15K) = 1.55 kcal/mol (2.11%)
ΔG(298.15K) = 1.46 kcal/mol (2.86%)
ΔG(216.65K) = 1.91 kcal/mol (0.50%)
ΔG(273.15K) = 1.74 kcal/mol (1.49%)
ΔG(298.15K) = 1.66 kcal/mol (2.03%)
ΔG(216.65K) = 1.62 kcal/mol (0.96%)
ΔG(273.15K) = 1.84 kcal/mol (1.21%)
ΔG(298.15K) = 1.94 kcal/mol (1.25%)
ΔG(216.65K) = 2.01 kcal/mol (0.36%)
ΔG(273.15K) = 1.83 kcal/mol (1.28%)
ΔG(298.15K) = 1.72 kcal/mol (1.86%)

## Slide 5
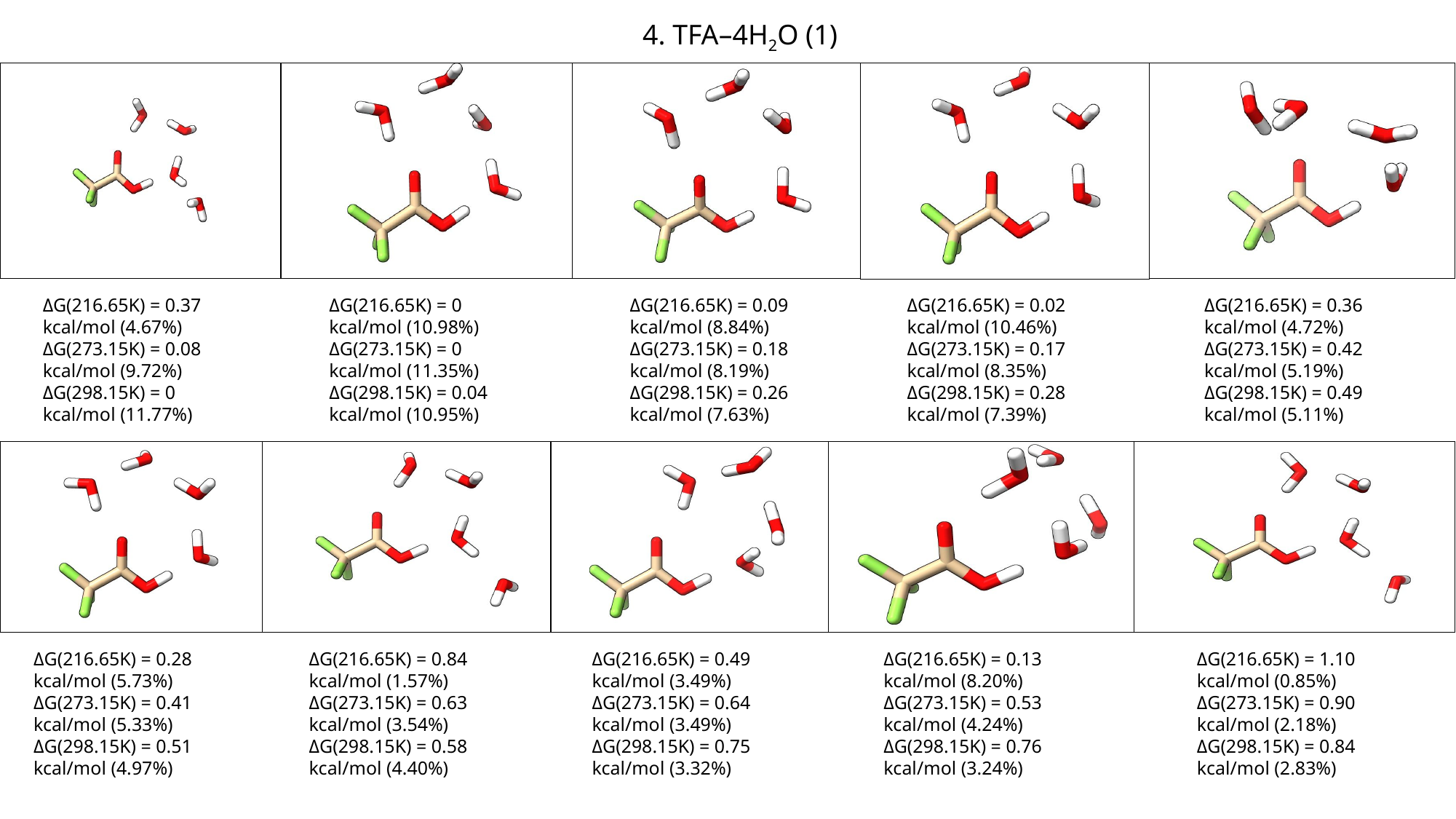

4. TFA–4H2O (1)
ΔG(216.65K) = 0.36 kcal/mol (4.72%)
ΔG(273.15K) = 0.42 kcal/mol (5.19%)
ΔG(298.15K) = 0.49 kcal/mol (5.11%)
ΔG(216.65K) = 0 kcal/mol (10.98%)
ΔG(273.15K) = 0 kcal/mol (11.35%)
ΔG(298.15K) = 0.04 kcal/mol (10.95%)
ΔG(216.65K) = 0.09 kcal/mol (8.84%)
ΔG(273.15K) = 0.18 kcal/mol (8.19%)
ΔG(298.15K) = 0.26 kcal/mol (7.63%)
ΔG(216.65K) = 0.02 kcal/mol (10.46%)
ΔG(273.15K) = 0.17 kcal/mol (8.35%)
ΔG(298.15K) = 0.28 kcal/mol (7.39%)
ΔG(216.65K) = 0.37 kcal/mol (4.67%)
ΔG(273.15K) = 0.08 kcal/mol (9.72%)
ΔG(298.15K) = 0 kcal/mol (11.77%)
ΔG(216.65K) = 0.84 kcal/mol (1.57%)
ΔG(273.15K) = 0.63 kcal/mol (3.54%)
ΔG(298.15K) = 0.58 kcal/mol (4.40%)
ΔG(216.65K) = 0.49 kcal/mol (3.49%)
ΔG(273.15K) = 0.64 kcal/mol (3.49%)
ΔG(298.15K) = 0.75 kcal/mol (3.32%)
ΔG(216.65K) = 0.13 kcal/mol (8.20%)
ΔG(273.15K) = 0.53 kcal/mol (4.24%)
ΔG(298.15K) = 0.76 kcal/mol (3.24%)
ΔG(216.65K) = 1.10 kcal/mol (0.85%)
ΔG(273.15K) = 0.90 kcal/mol (2.18%)
ΔG(298.15K) = 0.84 kcal/mol (2.83%)
ΔG(216.65K) = 0.28 kcal/mol (5.73%)
ΔG(273.15K) = 0.41 kcal/mol (5.33%)
ΔG(298.15K) = 0.51 kcal/mol (4.97%)

## Slide 6
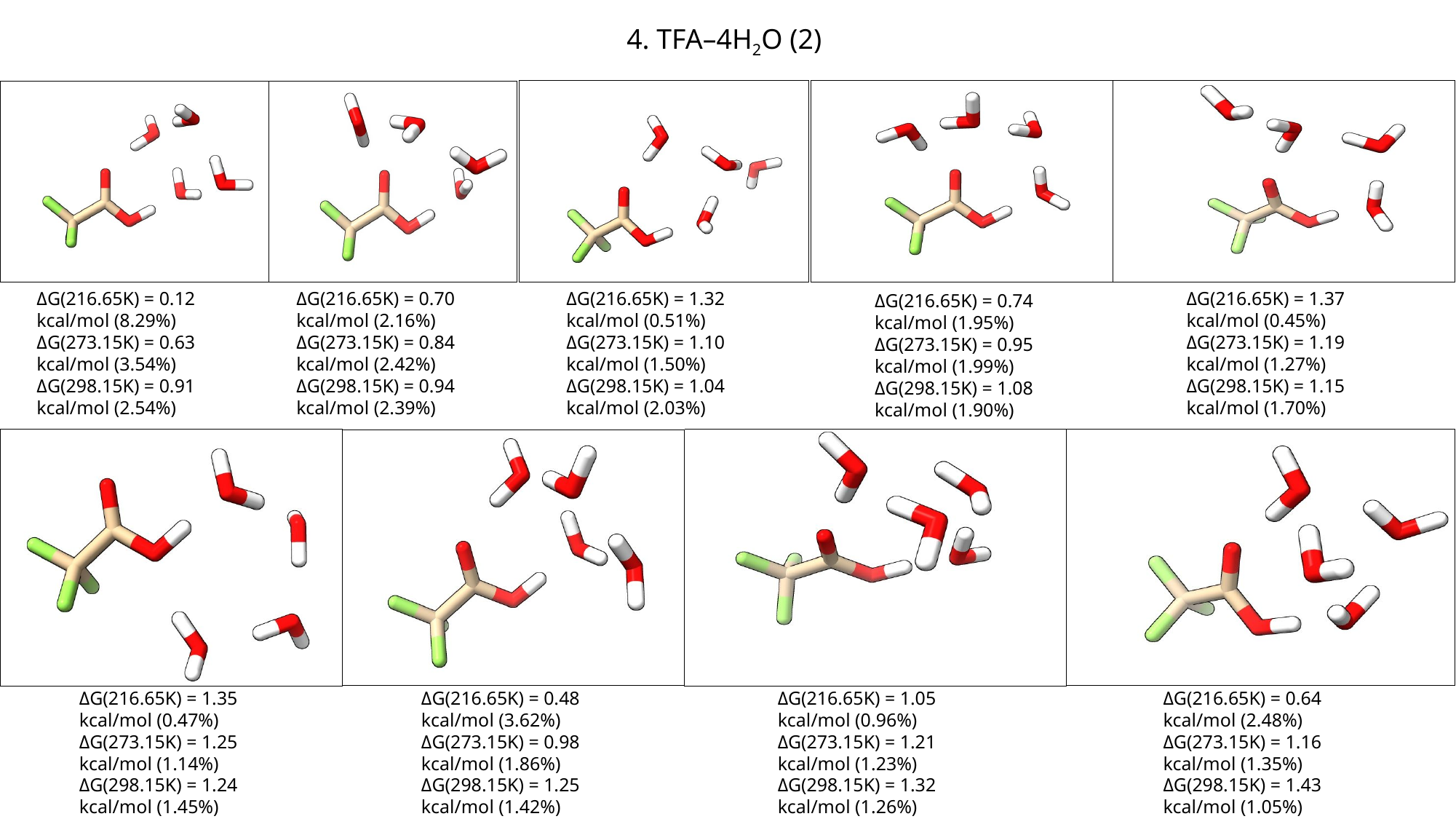

4. TFA–4H2O (2)
ΔG(216.65K) = 1.37 kcal/mol (0.45%)
ΔG(273.15K) = 1.19 kcal/mol (1.27%)
ΔG(298.15K) = 1.15 kcal/mol (1.70%)
ΔG(216.65K) = 1.32 kcal/mol (0.51%)
ΔG(273.15K) = 1.10 kcal/mol (1.50%)
ΔG(298.15K) = 1.04 kcal/mol (2.03%)
ΔG(216.65K) = 0.70 kcal/mol (2.16%)
ΔG(273.15K) = 0.84 kcal/mol (2.42%)
ΔG(298.15K) = 0.94 kcal/mol (2.39%)
ΔG(216.65K) = 0.12 kcal/mol (8.29%)
ΔG(273.15K) = 0.63 kcal/mol (3.54%)
ΔG(298.15K) = 0.91 kcal/mol (2.54%)
ΔG(216.65K) = 0.74 kcal/mol (1.95%)
ΔG(273.15K) = 0.95 kcal/mol (1.99%)
ΔG(298.15K) = 1.08 kcal/mol (1.90%)
ΔG(216.65K) = 1.05 kcal/mol (0.96%)
ΔG(273.15K) = 1.21 kcal/mol (1.23%)
ΔG(298.15K) = 1.32 kcal/mol (1.26%)
ΔG(216.65K) = 0.64 kcal/mol (2.48%)
ΔG(273.15K) = 1.16 kcal/mol (1.35%)
ΔG(298.15K) = 1.43 kcal/mol (1.05%)
ΔG(216.65K) = 1.35 kcal/mol (0.47%)
ΔG(273.15K) = 1.25 kcal/mol (1.14%)
ΔG(298.15K) = 1.24 kcal/mol (1.45%)
ΔG(216.65K) = 0.48 kcal/mol (3.62%)
ΔG(273.15K) = 0.98 kcal/mol (1.86%)
ΔG(298.15K) = 1.25 kcal/mol (1.42%)

## Slide 7
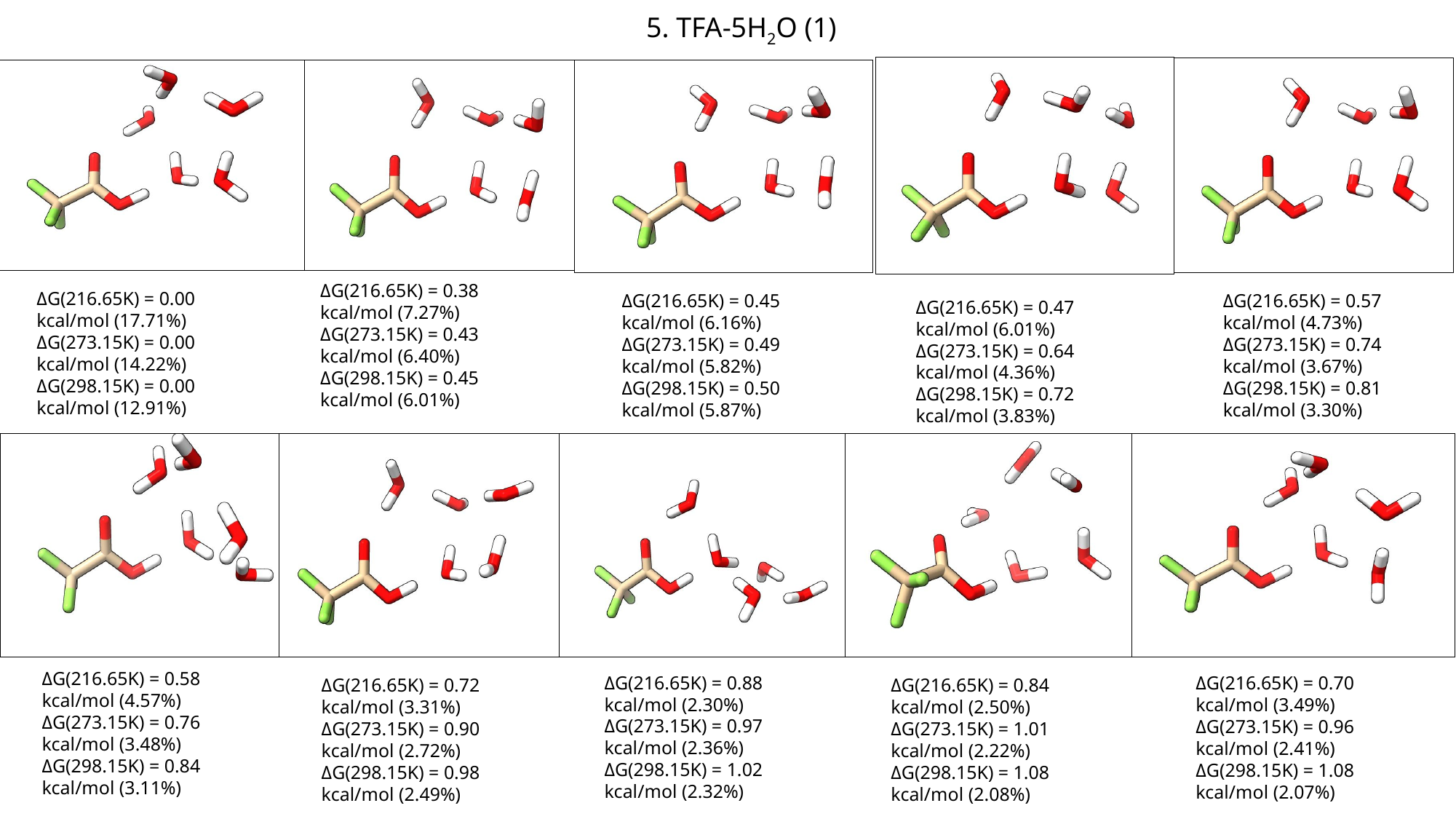

5. TFA-5H2O (1)
ΔG(216.65K) = 0.38 kcal/mol (7.27%)
ΔG(273.15K) = 0.43 kcal/mol (6.40%)
ΔG(298.15K) = 0.45 kcal/mol (6.01%)
ΔG(216.65K) = 0.00 kcal/mol (17.71%)
ΔG(273.15K) = 0.00 kcal/mol (14.22%)
ΔG(298.15K) = 0.00 kcal/mol (12.91%)
ΔG(216.65K) = 0.57 kcal/mol (4.73%)
ΔG(273.15K) = 0.74 kcal/mol (3.67%)
ΔG(298.15K) = 0.81 kcal/mol (3.30%)
ΔG(216.65K) = 0.45 kcal/mol (6.16%)
ΔG(273.15K) = 0.49 kcal/mol (5.82%)
ΔG(298.15K) = 0.50 kcal/mol (5.87%)
ΔG(216.65K) = 0.47 kcal/mol (6.01%)
ΔG(273.15K) = 0.64 kcal/mol (4.36%)
ΔG(298.15K) = 0.72 kcal/mol (3.83%)
ΔG(216.65K) = 0.58 kcal/mol (4.57%)
ΔG(273.15K) = 0.76 kcal/mol (3.48%)
ΔG(298.15K) = 0.84 kcal/mol (3.11%)
ΔG(216.65K) = 0.88 kcal/mol (2.30%)
ΔG(273.15K) = 0.97 kcal/mol (2.36%)
ΔG(298.15K) = 1.02 kcal/mol (2.32%)
ΔG(216.65K) = 0.70 kcal/mol (3.49%)
ΔG(273.15K) = 0.96 kcal/mol (2.41%)
ΔG(298.15K) = 1.08 kcal/mol (2.07%)
ΔG(216.65K) = 0.72 kcal/mol (3.31%)
ΔG(273.15K) = 0.90 kcal/mol (2.72%)
ΔG(298.15K) = 0.98 kcal/mol (2.49%)
ΔG(216.65K) = 0.84 kcal/mol (2.50%)
ΔG(273.15K) = 1.01 kcal/mol (2.22%)
ΔG(298.15K) = 1.08 kcal/mol (2.08%)

## Slide 8
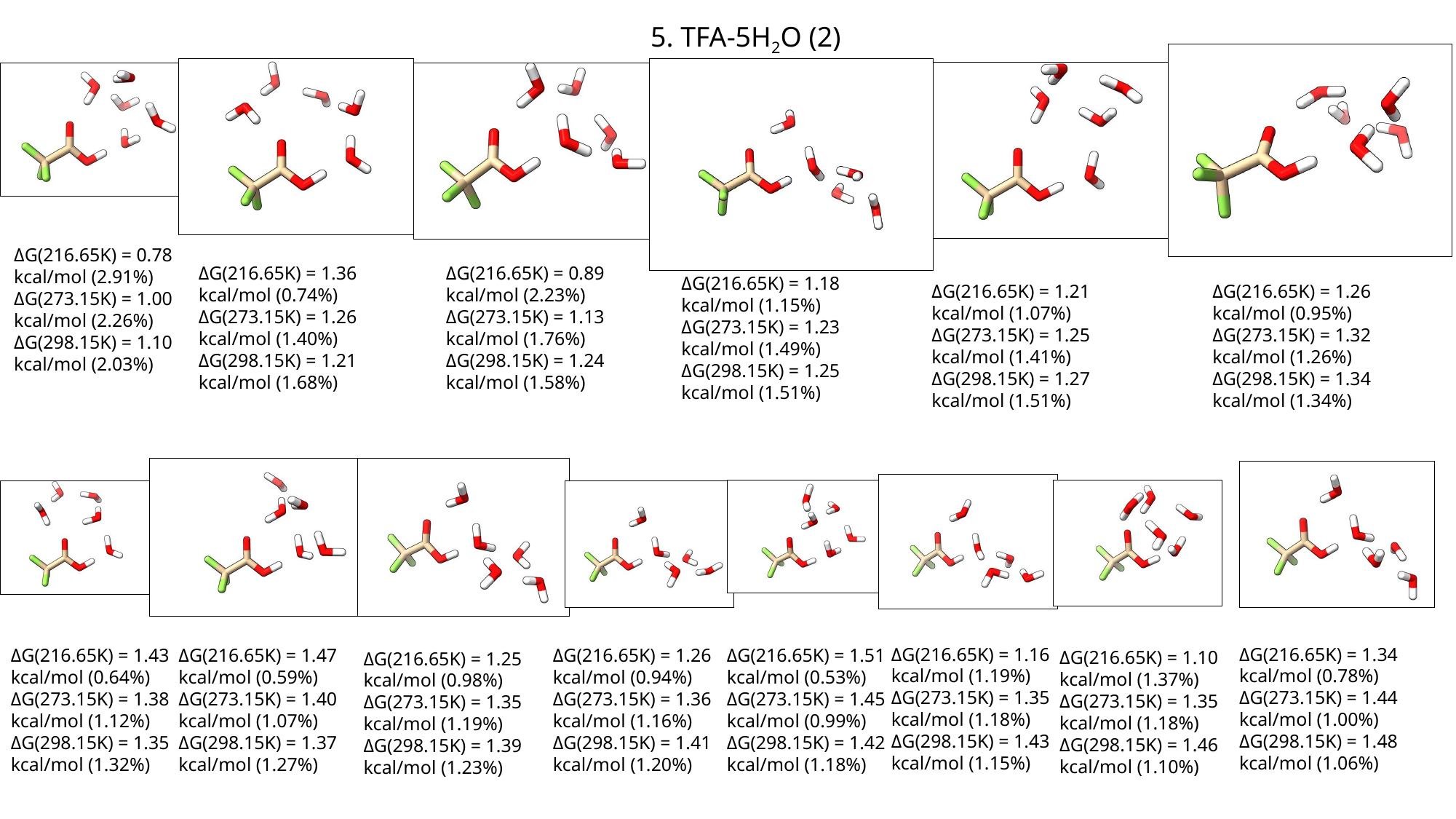

5. TFA-5H2O (2)
ΔG(216.65K) = 0.78 kcal/mol (2.91%)
ΔG(273.15K) = 1.00 kcal/mol (2.26%)
ΔG(298.15K) = 1.10 kcal/mol (2.03%)
ΔG(216.65K) = 1.36 kcal/mol (0.74%)
ΔG(273.15K) = 1.26 kcal/mol (1.40%)
ΔG(298.15K) = 1.21 kcal/mol (1.68%)
ΔG(216.65K) = 0.89 kcal/mol (2.23%)
ΔG(273.15K) = 1.13 kcal/mol (1.76%)
ΔG(298.15K) = 1.24 kcal/mol (1.58%)
ΔG(216.65K) = 1.18 kcal/mol (1.15%)
ΔG(273.15K) = 1.23 kcal/mol (1.49%)
ΔG(298.15K) = 1.25 kcal/mol (1.51%)
ΔG(216.65K) = 1.21 kcal/mol (1.07%)
ΔG(273.15K) = 1.25 kcal/mol (1.41%)
ΔG(298.15K) = 1.27 kcal/mol (1.51%)
ΔG(216.65K) = 1.26 kcal/mol (0.95%)
ΔG(273.15K) = 1.32 kcal/mol (1.26%)
ΔG(298.15K) = 1.34 kcal/mol (1.34%)
ΔG(216.65K) = 1.16 kcal/mol (1.19%)
ΔG(273.15K) = 1.35 kcal/mol (1.18%)
ΔG(298.15K) = 1.43 kcal/mol (1.15%)
ΔG(216.65K) = 1.34 kcal/mol (0.78%)
ΔG(273.15K) = 1.44 kcal/mol (1.00%)
ΔG(298.15K) = 1.48 kcal/mol (1.06%)
ΔG(216.65K) = 1.26 kcal/mol (0.94%)
ΔG(273.15K) = 1.36 kcal/mol (1.16%)
ΔG(298.15K) = 1.41 kcal/mol (1.20%)
ΔG(216.65K) = 1.51 kcal/mol (0.53%)
ΔG(273.15K) = 1.45 kcal/mol (0.99%)
ΔG(298.15K) = 1.42 kcal/mol (1.18%)
ΔG(216.65K) = 1.43 kcal/mol (0.64%)
ΔG(273.15K) = 1.38 kcal/mol (1.12%)
ΔG(298.15K) = 1.35 kcal/mol (1.32%)
ΔG(216.65K) = 1.47 kcal/mol (0.59%)
ΔG(273.15K) = 1.40 kcal/mol (1.07%)
ΔG(298.15K) = 1.37 kcal/mol (1.27%)
ΔG(216.65K) = 1.10 kcal/mol (1.37%)
ΔG(273.15K) = 1.35 kcal/mol (1.18%)
ΔG(298.15K) = 1.46 kcal/mol (1.10%)
ΔG(216.65K) = 1.25 kcal/mol (0.98%)
ΔG(273.15K) = 1.35 kcal/mol (1.19%)
ΔG(298.15K) = 1.39 kcal/mol (1.23%)

## Slide 9
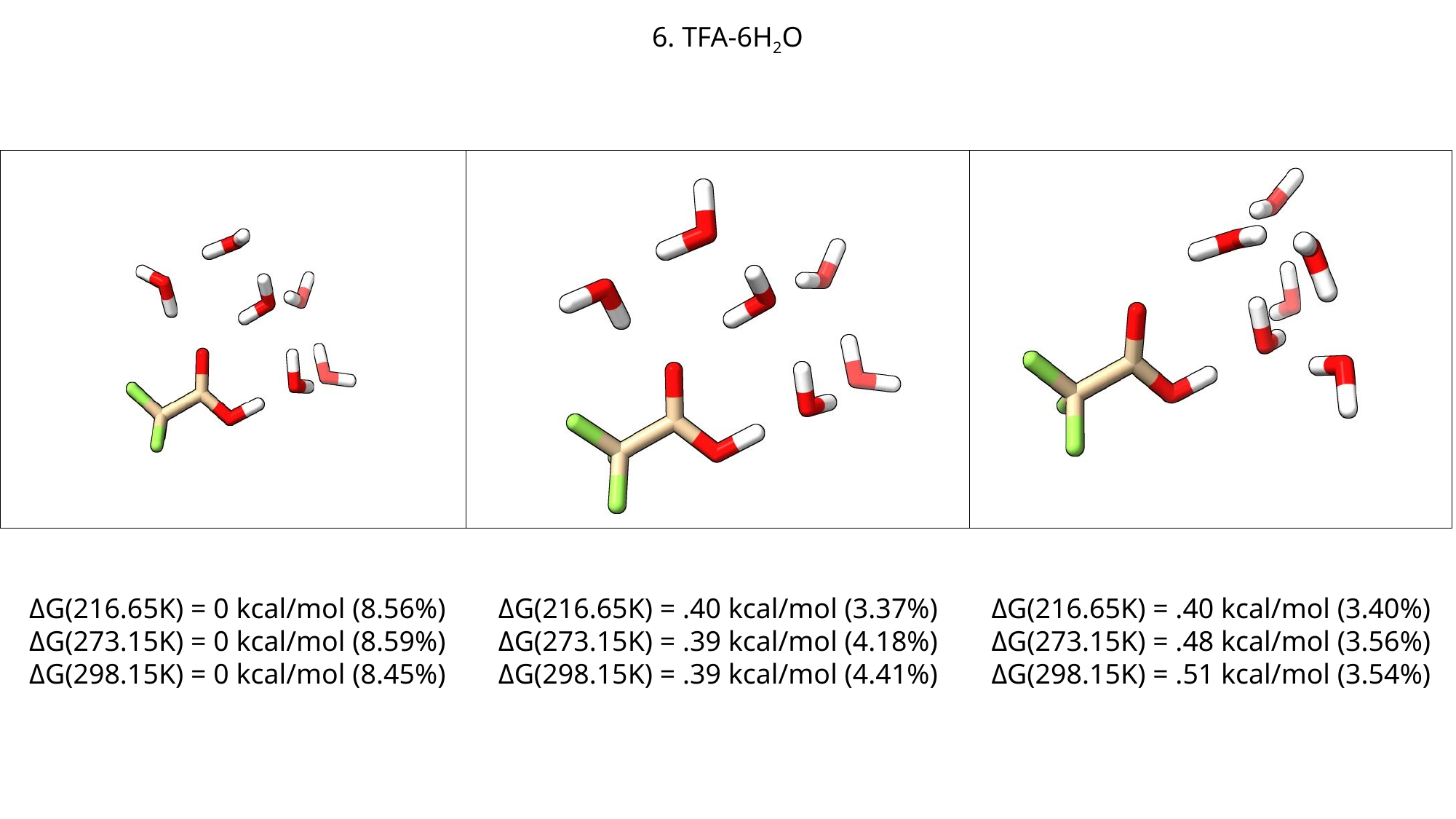

6. TFA-6H2O
ΔG(216.65K) = 0 kcal/mol (8.56%)
ΔG(273.15K) = 0 kcal/mol (8.59%)
ΔG(298.15K) = 0 kcal/mol (8.45%)
ΔG(216.65K) = .40 kcal/mol (3.37%)
ΔG(273.15K) = .39 kcal/mol (4.18%)
ΔG(298.15K) = .39 kcal/mol (4.41%)
ΔG(216.65K) = .40 kcal/mol (3.40%)
ΔG(273.15K) = .48 kcal/mol (3.56%)
ΔG(298.15K) = .51 kcal/mol (3.54%)

## Slide 10
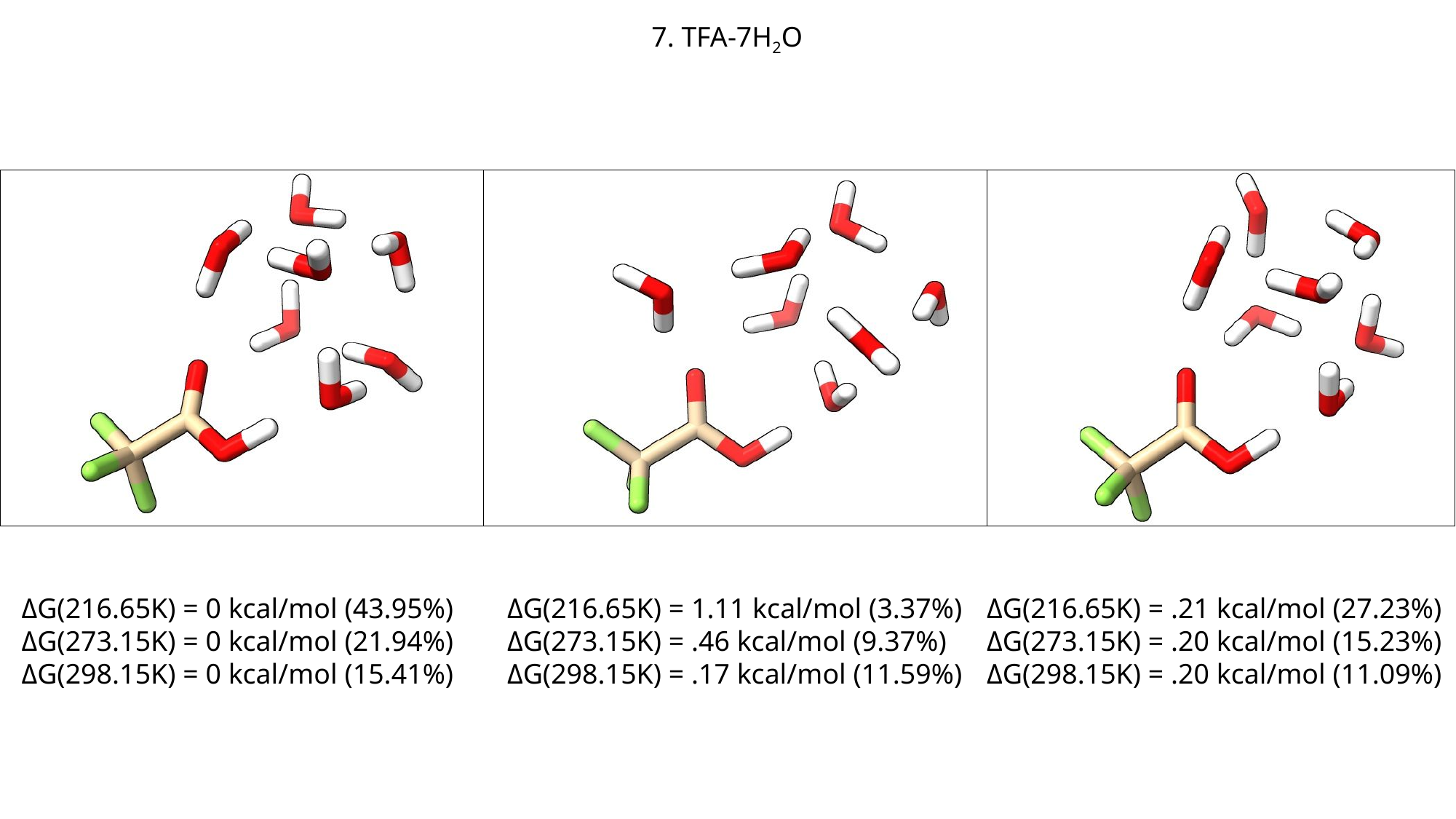

7. TFA-7H2O
ΔG(216.65K) = 0 kcal/mol (43.95%)
ΔG(273.15K) = 0 kcal/mol (21.94%)
ΔG(298.15K) = 0 kcal/mol (15.41%)
ΔG(216.65K) = 1.11 kcal/mol (3.37%)
ΔG(273.15K) = .46 kcal/mol (9.37%)
ΔG(298.15K) = .17 kcal/mol (11.59%)
ΔG(216.65K) = .21 kcal/mol (27.23%)
ΔG(273.15K) = .20 kcal/mol (15.23%)
ΔG(298.15K) = .20 kcal/mol (11.09%)

## Slide 11
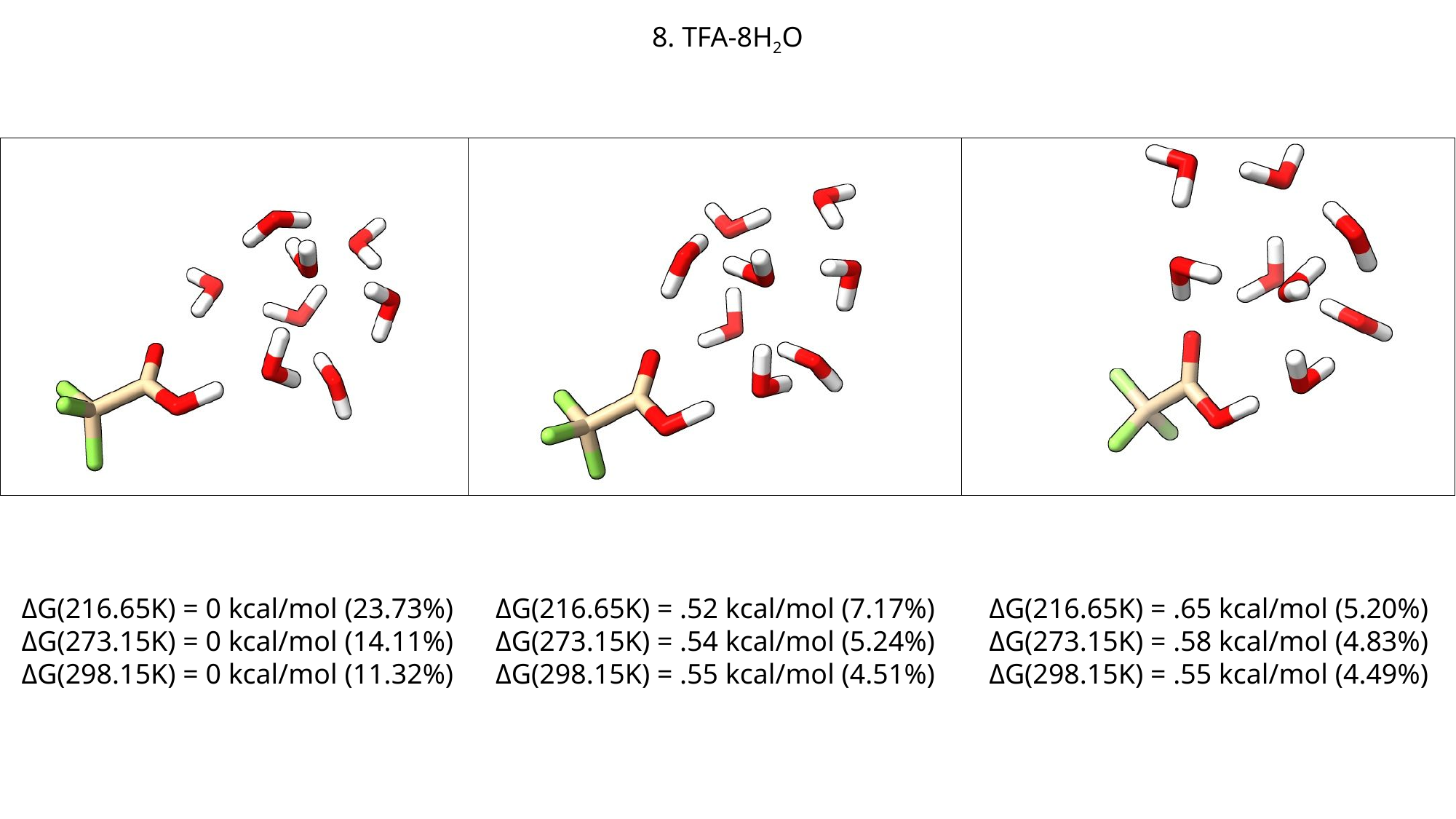

8. TFA-8H2O
ΔG(216.65K) = 0 kcal/mol (23.73%)
ΔG(273.15K) = 0 kcal/mol (14.11%)
ΔG(298.15K) = 0 kcal/mol (11.32%)
ΔG(216.65K) = .52 kcal/mol (7.17%)
ΔG(273.15K) = .54 kcal/mol (5.24%)
ΔG(298.15K) = .55 kcal/mol (4.51%)
ΔG(216.65K) = .65 kcal/mol (5.20%)
ΔG(273.15K) = .58 kcal/mol (4.83%)
ΔG(298.15K) = .55 kcal/mol (4.49%)
