# Supplementary material for: The Hydration of Trifluoroacetic Acid from 0 to 298 K
Source: J Phys Chem A. 2026 Jan 20;130(4):927–35. doi: 10.1021/acs.jpca.5c08151 (PMC12862820; doi:10.1021/acs.jpca.5c08151)
Supplement: Supplementary file 1 [file jp5c08151_si_001.zip › TFA_SI/Figures 0 K.pptx]

## Slide 1
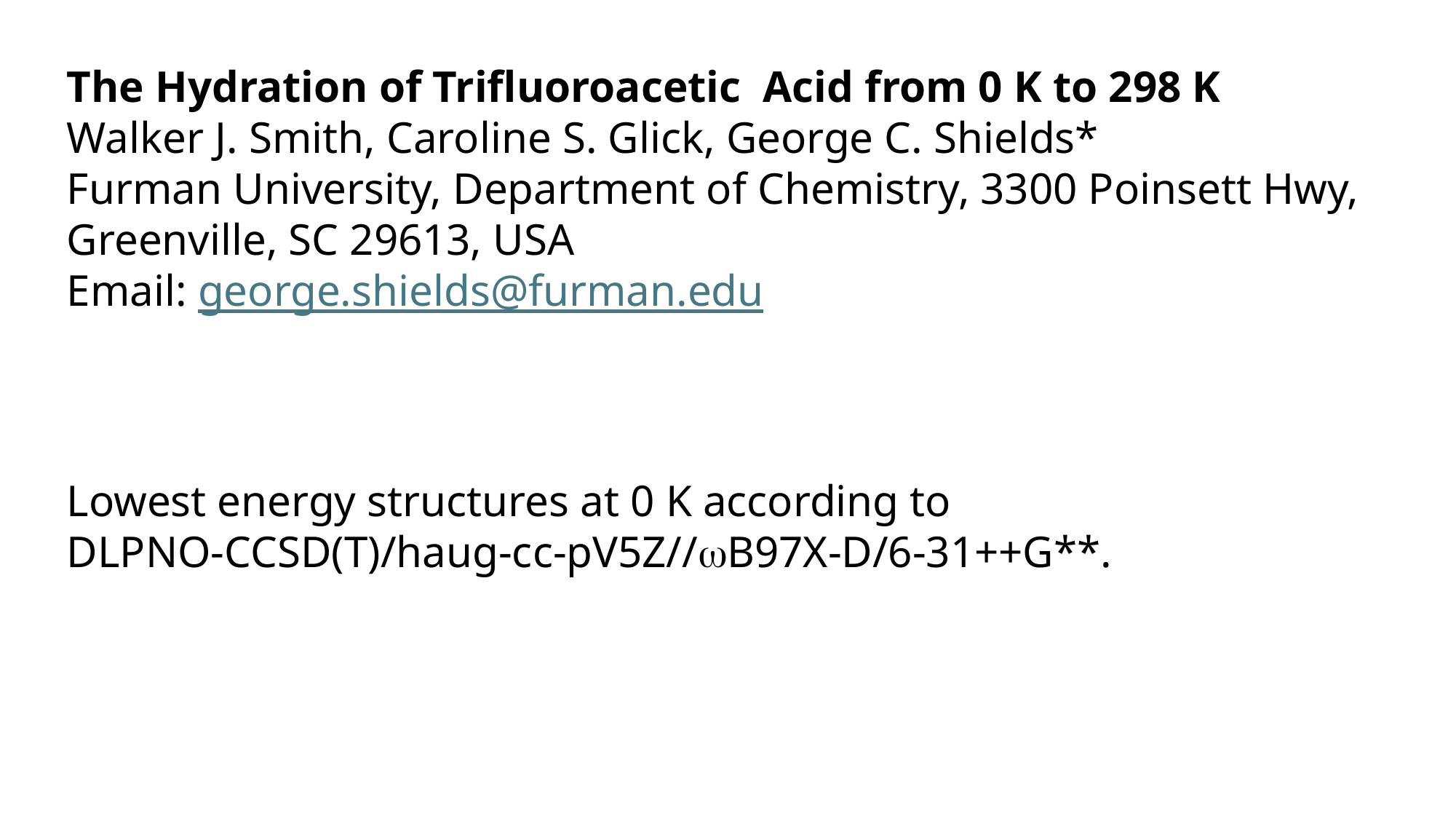

The Hydration of Trifluoroacetic  Acid from 0 K to 298 K
Walker J. Smith, Caroline S. Glick, George C. Shields*
Furman University, Department of Chemistry, 3300 Poinsett Hwy, Greenville, SC 29613, USA
Email: george.shields@furman.edu
Lowest energy structures at 0 K according to DLPNO-CCSD(T)/haug-cc-pV5Z//ωB97X-D/6-31++G**.

## Slide 2
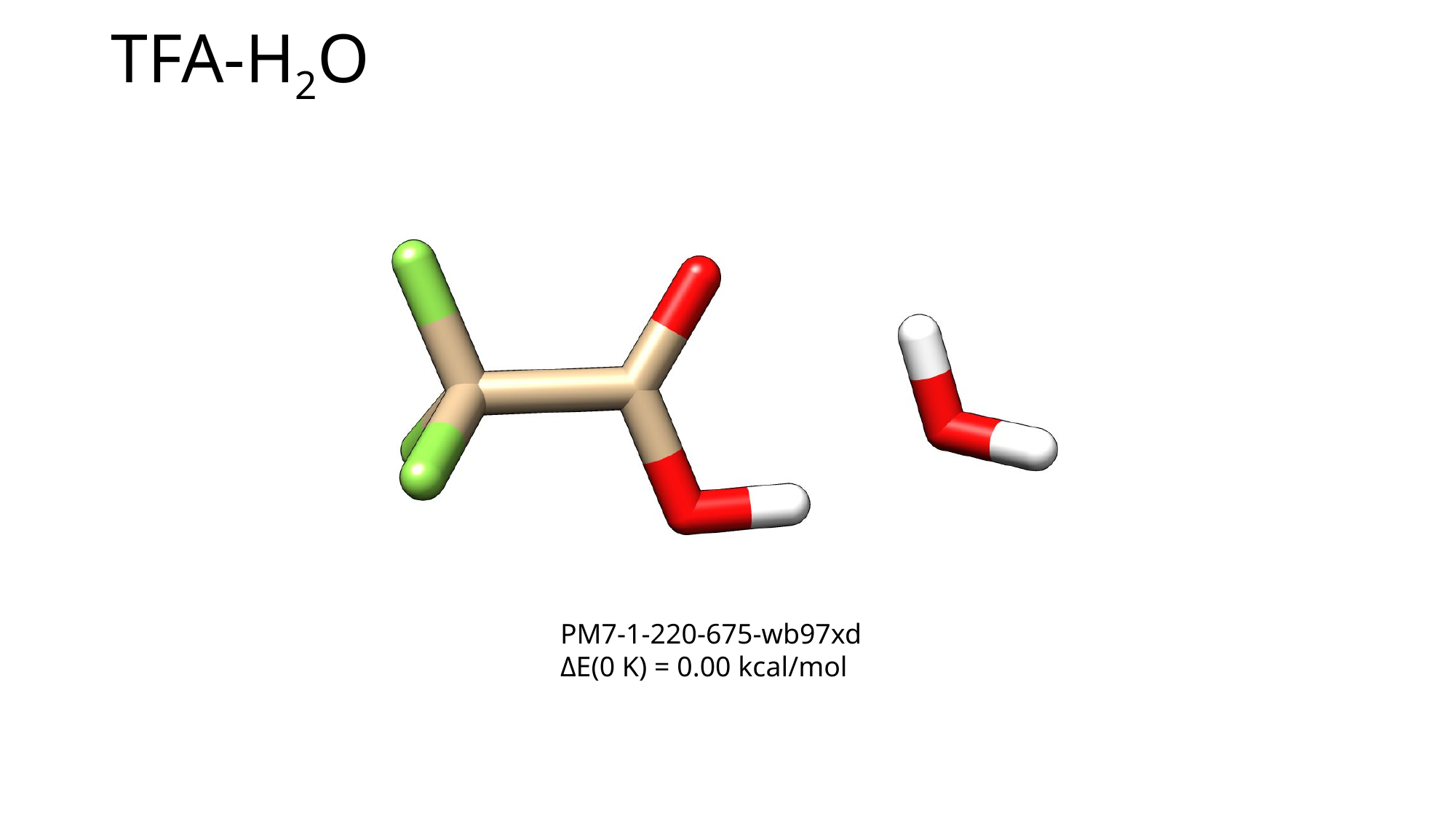

# TFA-H2O
PM7-1-220-675-wb97xd
ΔE(0 K) = 0.00 kcal/mol

## Slide 3
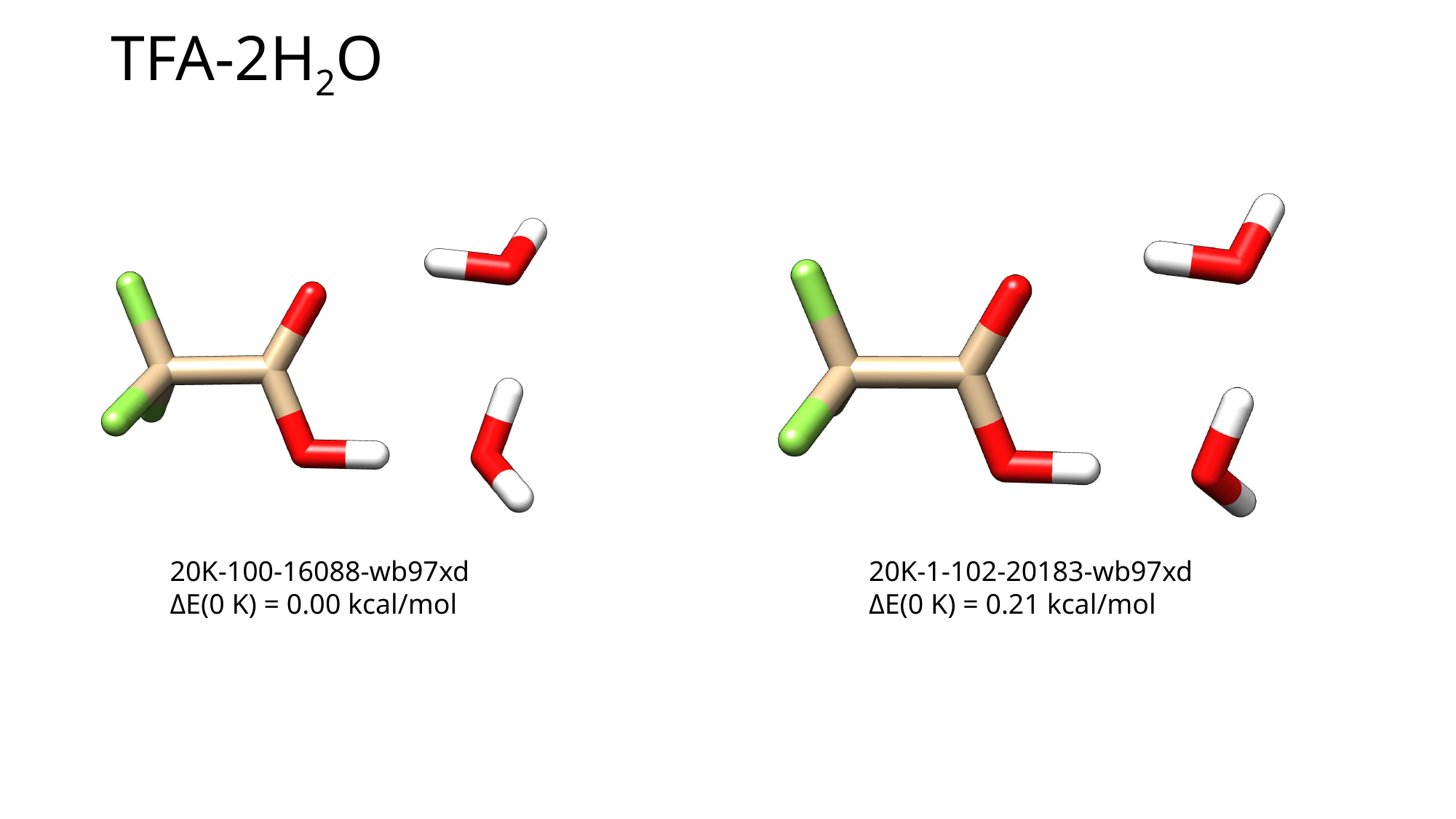

TFA-2H2O
20K-100-16088-wb97xd
ΔE(0 K) = 0.00 kcal/mol
20K-1-102-20183-wb97xd
ΔE(0 K) = 0.21 kcal/mol

## Slide 4
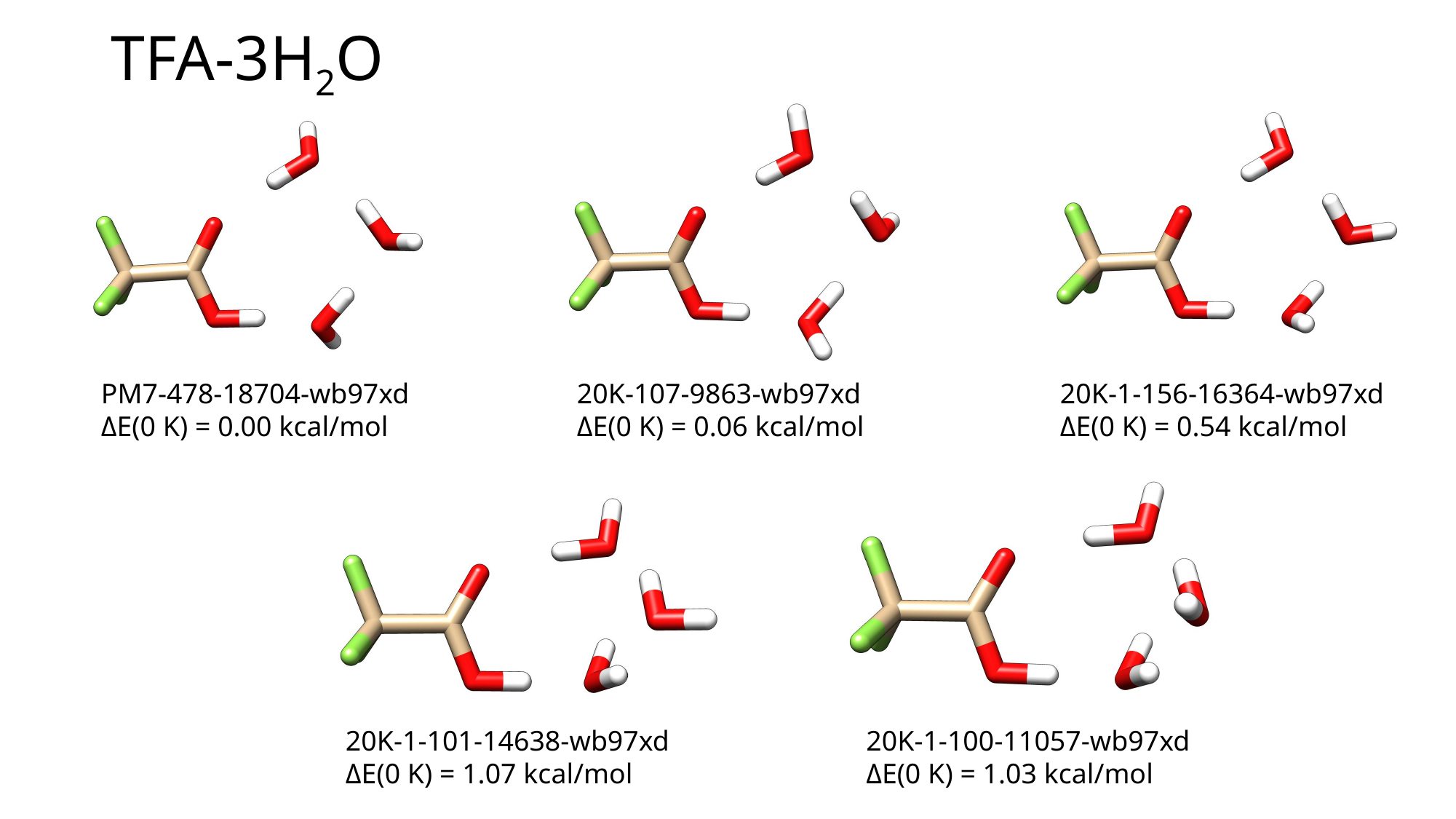

TFA-3H2O
20K-107-9863-wb97xd
ΔE(0 K) = 0.06 kcal/mol
20K-1-156-16364-wb97xd
ΔE(0 K) = 0.54 kcal/mol
PM7-478-18704-wb97xd
ΔE(0 K) = 0.00 kcal/mol
20K-1-101-14638-wb97xd
ΔE(0 K) = 1.07 kcal/mol
20K-1-100-11057-wb97xd
ΔE(0 K) = 1.03 kcal/mol

## Slide 5
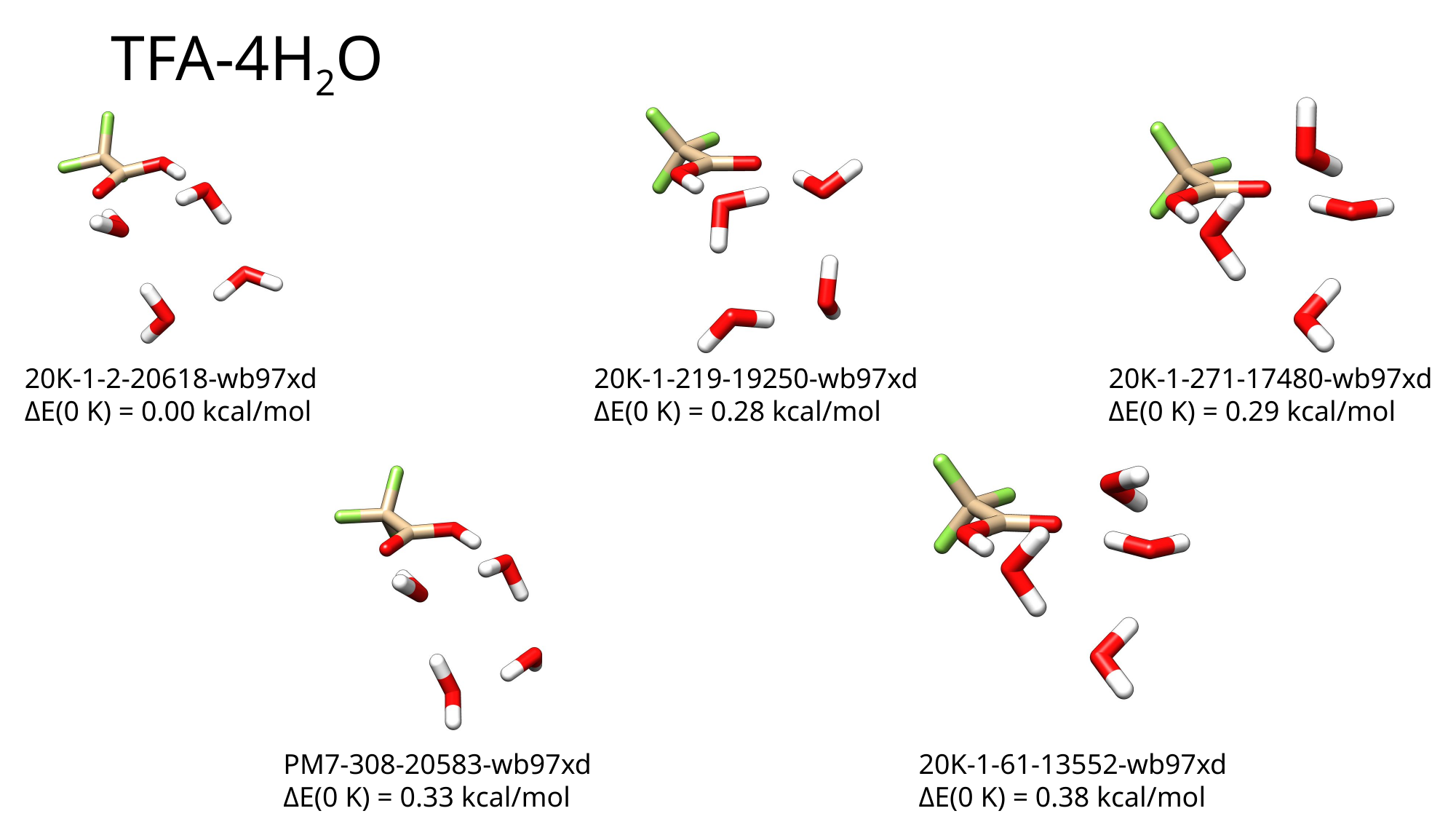

TFA-4H2O
20K-1-2-20618-wb97xd
ΔE(0 K) = 0.00 kcal/mol
20K-1-219-19250-wb97xd
ΔE(0 K) = 0.28 kcal/mol
20K-1-271-17480-wb97xd
ΔE(0 K) = 0.29 kcal/mol
PM7-308-20583-wb97xd
ΔE(0 K) = 0.33 kcal/mol
20K-1-61-13552-wb97xd
ΔE(0 K) = 0.38 kcal/mol

## Slide 6
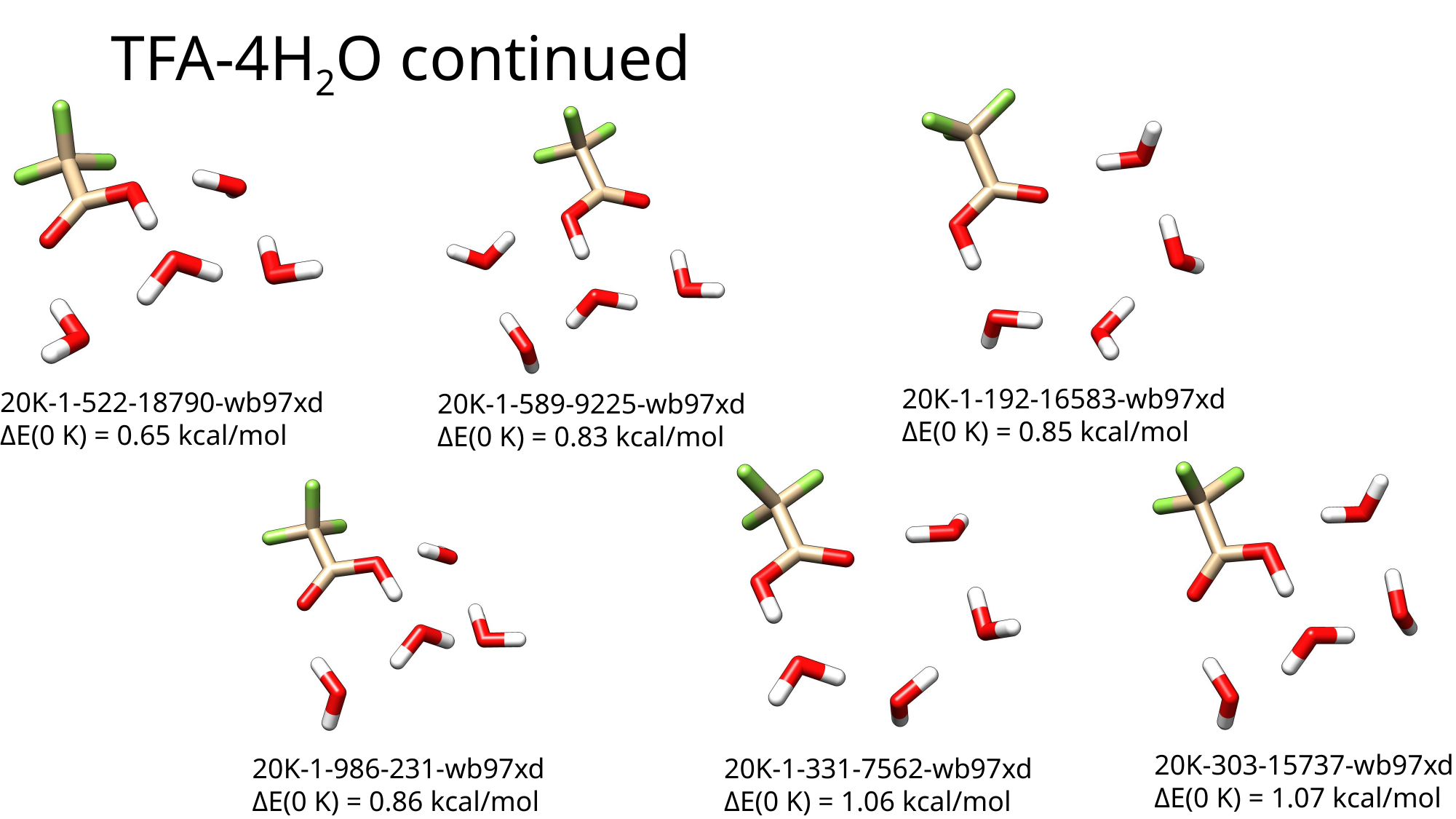

TFA-4H2O continued
20K-1-192-16583-wb97xd
ΔE(0 K) = 0.85 kcal/mol
20K-1-522-18790-wb97xd
ΔE(0 K) = 0.65 kcal/mol
20K-1-589-9225-wb97xd
ΔE(0 K) = 0.83 kcal/mol
20K-303-15737-wb97xd
ΔE(0 K) = 1.07 kcal/mol
20K-1-986-231-wb97xd
ΔE(0 K) = 0.86 kcal/mol
20K-1-331-7562-wb97xd
ΔE(0 K) = 1.06 kcal/mol

## Slide 7
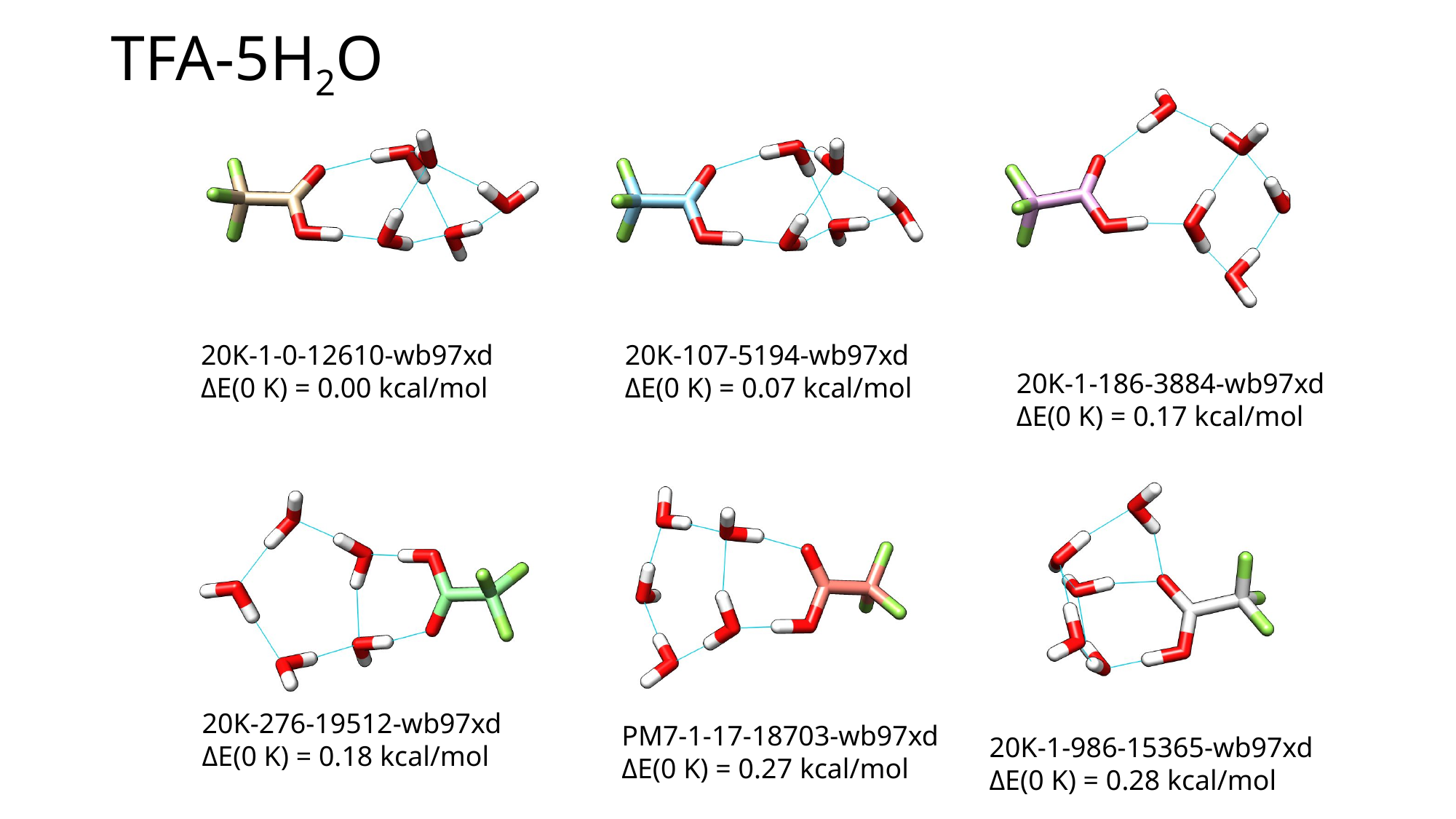

TFA-5H2O
20K-107-5194-wb97xd
ΔE(0 K) = 0.07 kcal/mol
20K-1-0-12610-wb97xd
ΔE(0 K) = 0.00 kcal/mol
20K-1-186-3884-wb97xd
ΔE(0 K) = 0.17 kcal/mol
20K-276-19512-wb97xd
ΔE(0 K) = 0.18 kcal/mol
PM7-1-17-18703-wb97xd
ΔE(0 K) = 0.27 kcal/mol
20K-1-986-15365-wb97xd
ΔE(0 K) = 0.28 kcal/mol

## Slide 8
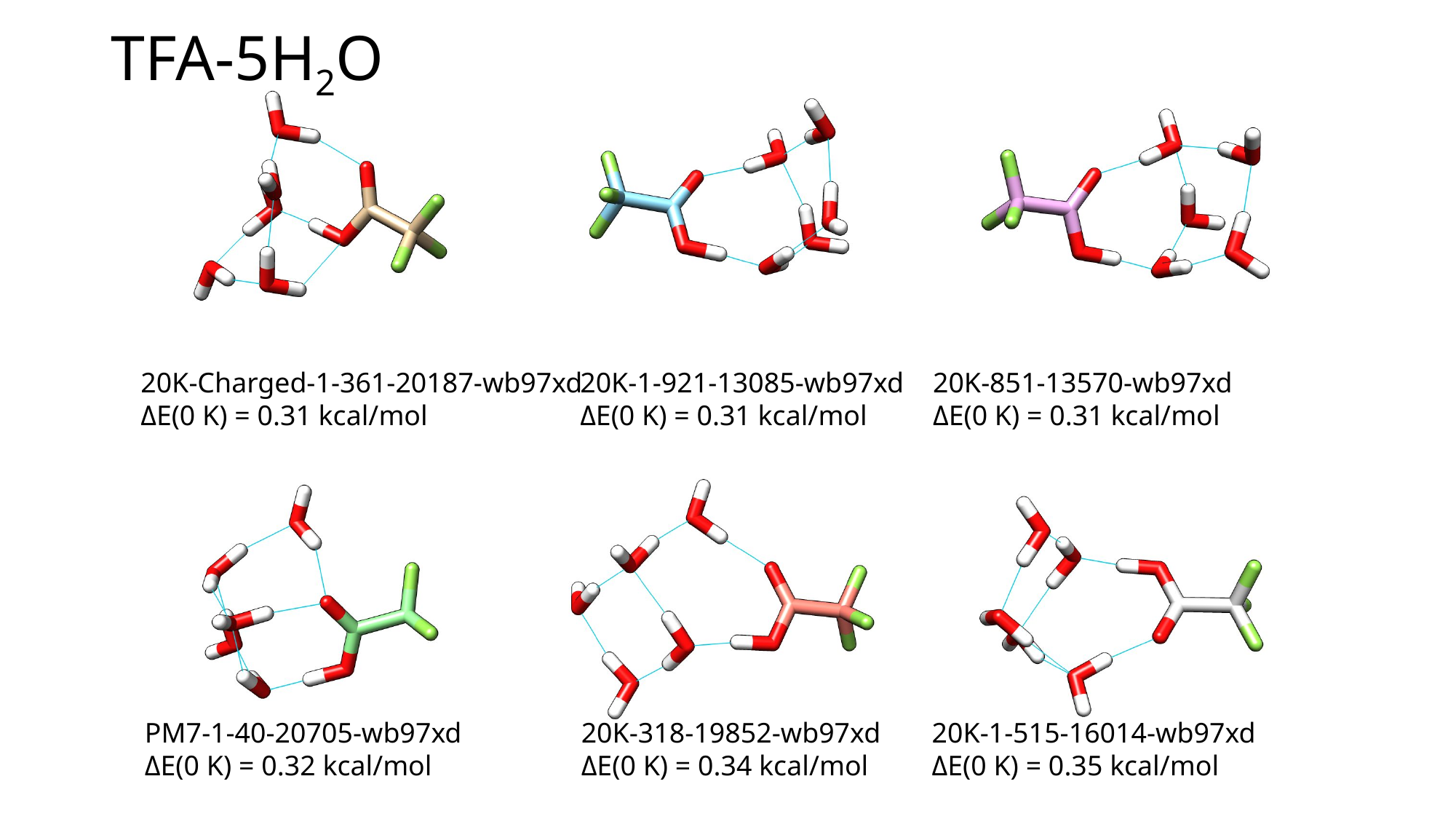

TFA-5H2O
20K-851-13570-wb97xd
ΔE(0 K) = 0.31 kcal/mol
20K-1-921-13085-wb97xd
ΔE(0 K) = 0.31 kcal/mol
20K-Charged-1-361-20187-wb97xd
ΔE(0 K) = 0.31 kcal/mol
20K-1-515-16014-wb97xd
ΔE(0 K) = 0.35 kcal/mol
20K-318-19852-wb97xd
ΔE(0 K) = 0.34 kcal/mol
PM7-1-40-20705-wb97xd
ΔE(0 K) = 0.32 kcal/mol

## Slide 9
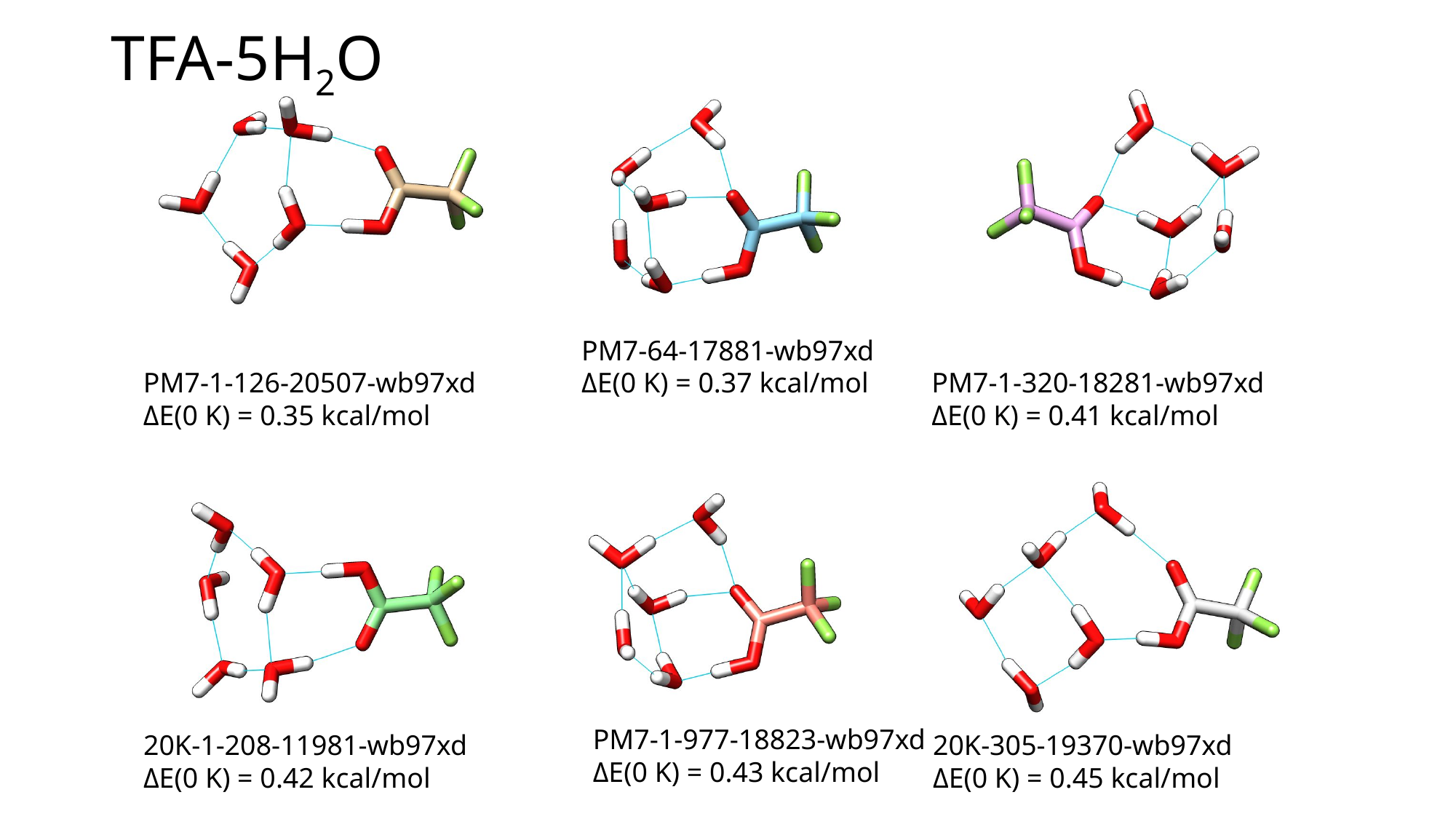

TFA-5H2O
PM7-64-17881-wb97xd
ΔE(0 K) = 0.37 kcal/mol
PM7-1-320-18281-wb97xd
ΔE(0 K) = 0.41 kcal/mol
PM7-1-126-20507-wb97xd
ΔE(0 K) = 0.35 kcal/mol
PM7-1-977-18823-wb97xd
ΔE(0 K) = 0.43 kcal/mol
20K-305-19370-wb97xd
ΔE(0 K) = 0.45 kcal/mol
20K-1-208-11981-wb97xd
ΔE(0 K) = 0.42 kcal/mol

## Slide 10
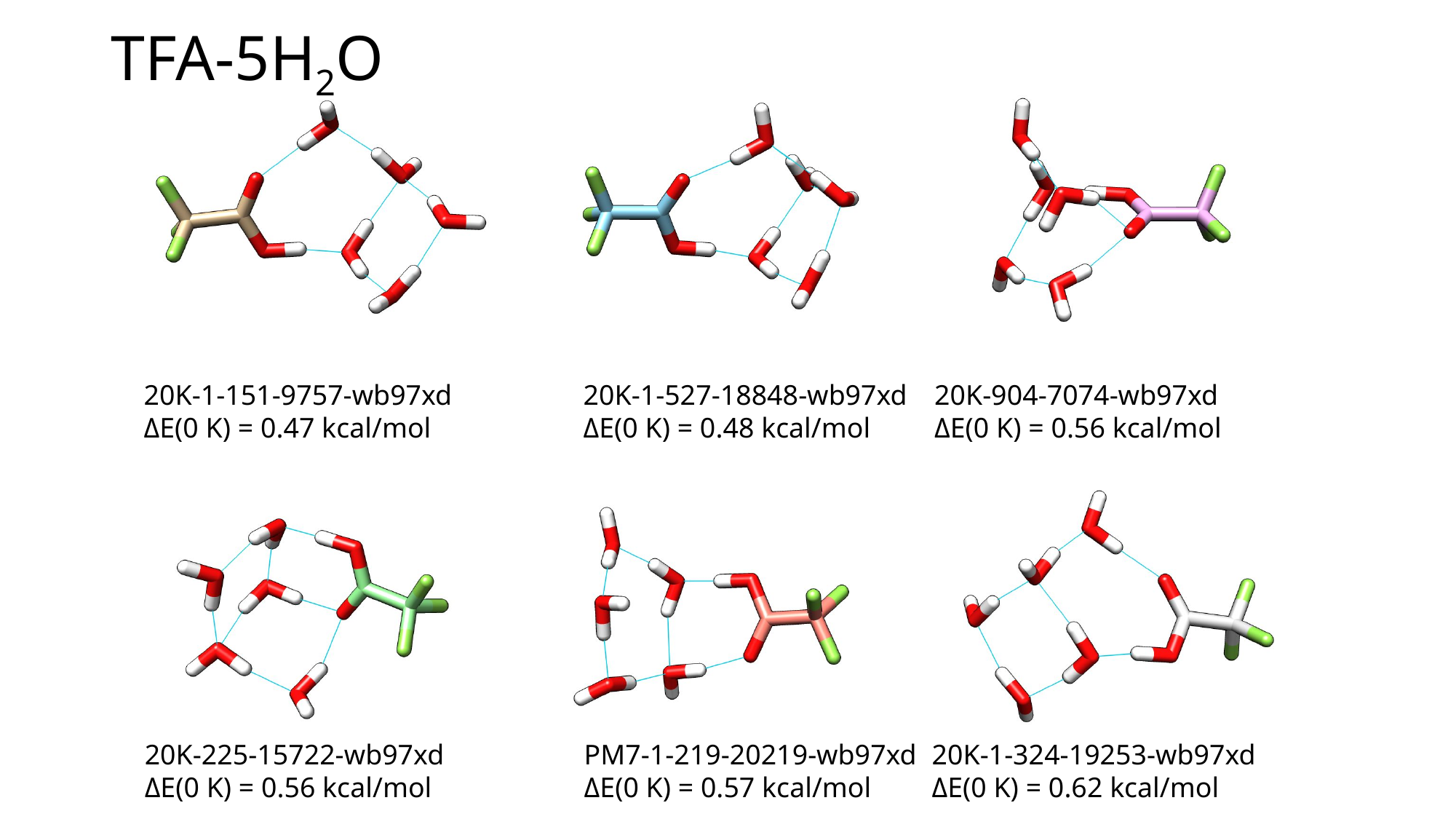

TFA-5H2O
20K-1-527-18848-wb97xd
ΔE(0 K) = 0.48 kcal/mol
20K-904-7074-wb97xd
ΔE(0 K) = 0.56 kcal/mol
20K-1-151-9757-wb97xd
ΔE(0 K) = 0.47 kcal/mol
PM7-1-219-20219-wb97xd
ΔE(0 K) = 0.57 kcal/mol
20K-1-324-19253-wb97xd
ΔE(0 K) = 0.62 kcal/mol
20K-225-15722-wb97xd
ΔE(0 K) = 0.56 kcal/mol

## Slide 11
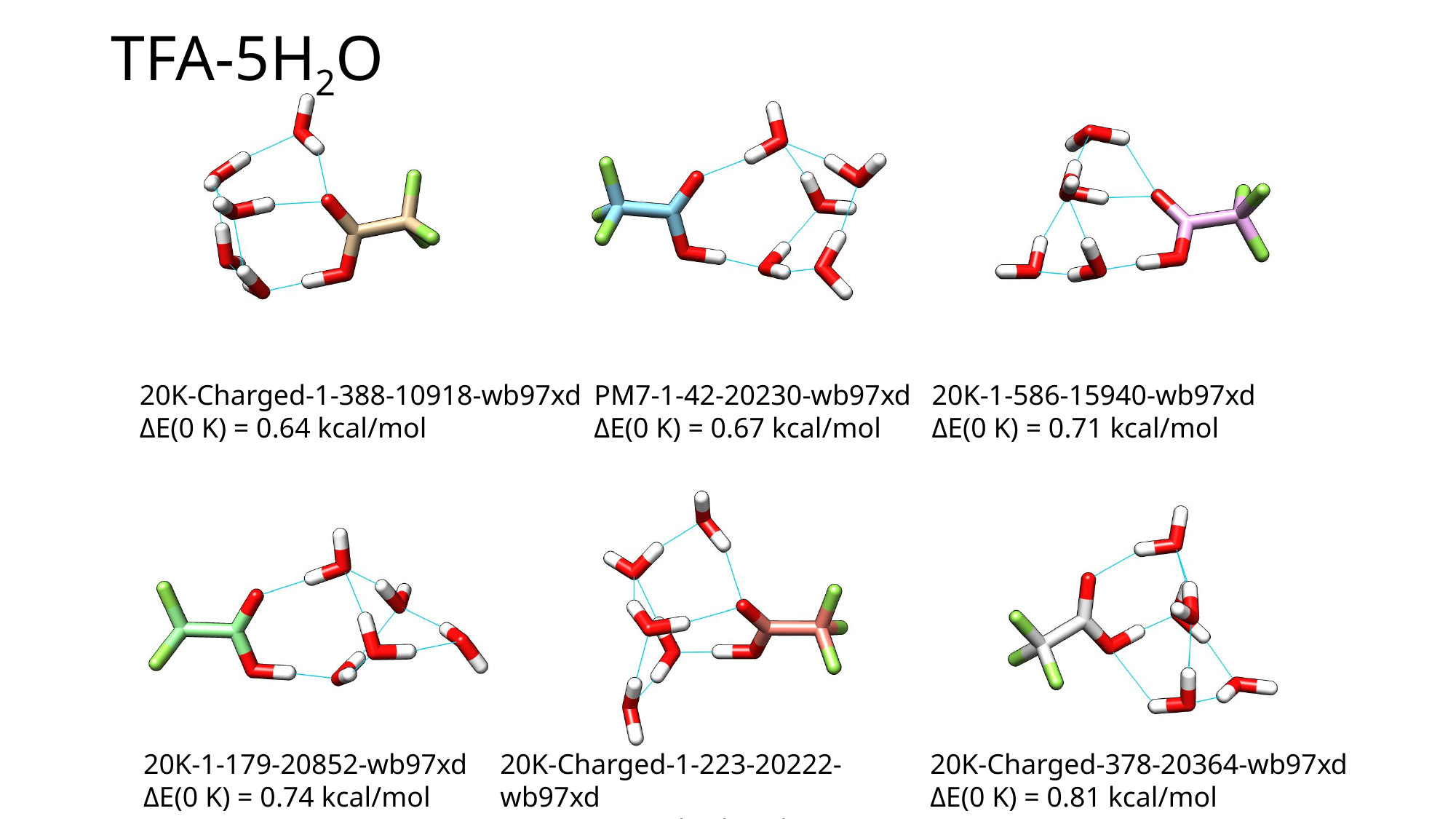

TFA-5H2O
PM7-1-42-20230-wb97xd
ΔE(0 K) = 0.67 kcal/mol
20K-1-586-15940-wb97xd
ΔE(0 K) = 0.71 kcal/mol
20K-Charged-1-388-10918-wb97xd
ΔE(0 K) = 0.64 kcal/mol
20K-Charged-1-223-20222-wb97xd
ΔE(0 K) = 0.79 kcal/mol
20K-Charged-378-20364-wb97xd
ΔE(0 K) = 0.81 kcal/mol
20K-1-179-20852-wb97xd
ΔE(0 K) = 0.74 kcal/mol

## Slide 12
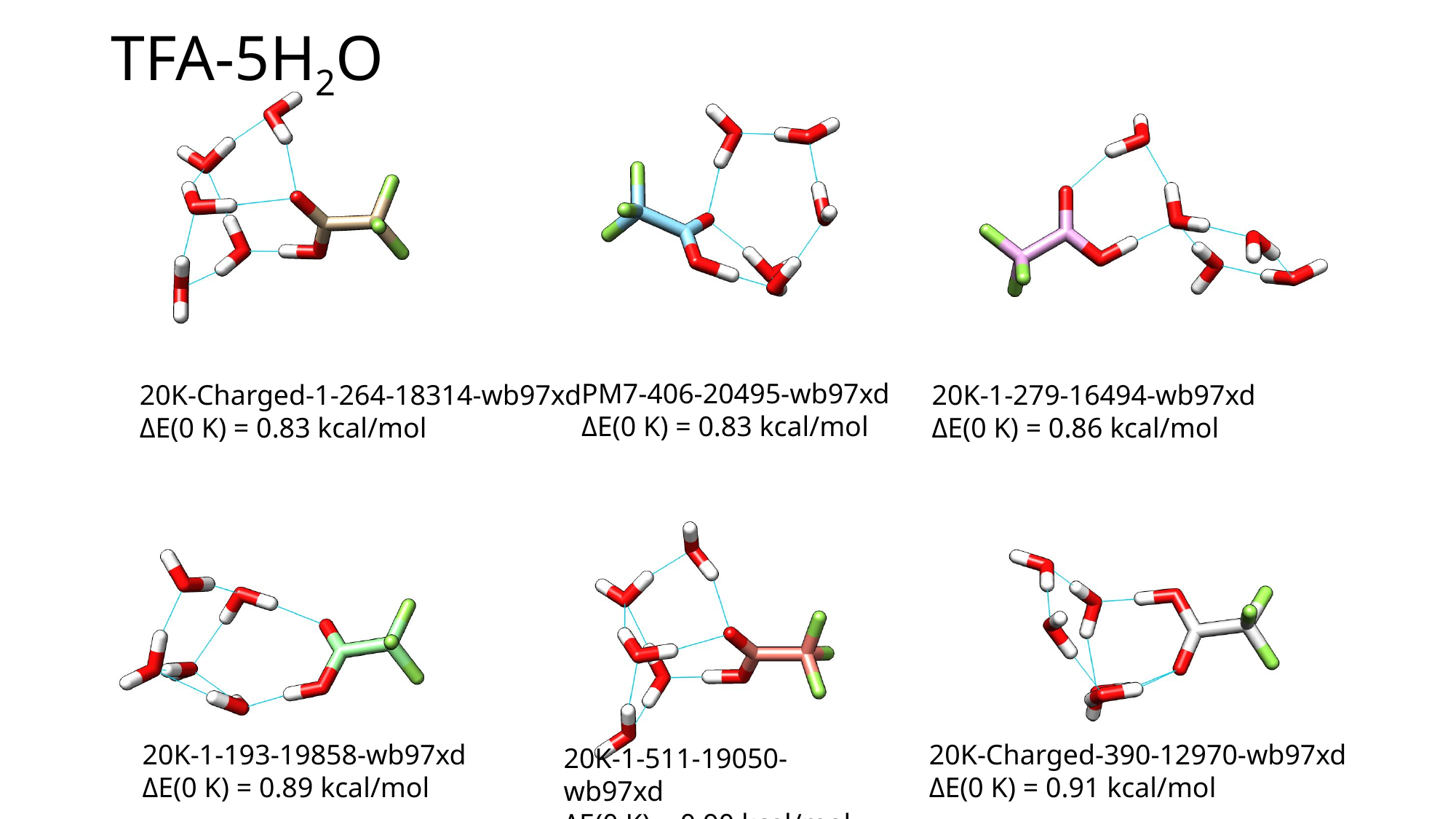

TFA-5H2O
PM7-406-20495-wb97xd
ΔE(0 K) = 0.83 kcal/mol
20K-1-279-16494-wb97xd
ΔE(0 K) = 0.86 kcal/mol
20K-Charged-1-264-18314-wb97xd
ΔE(0 K) = 0.83 kcal/mol
20K-Charged-390-12970-wb97xd
ΔE(0 K) = 0.91 kcal/mol
20K-1-193-19858-wb97xd
ΔE(0 K) = 0.89 kcal/mol
20K-1-511-19050-wb97xd
ΔE(0 K) = 0.90 kcal/mol

## Slide 13
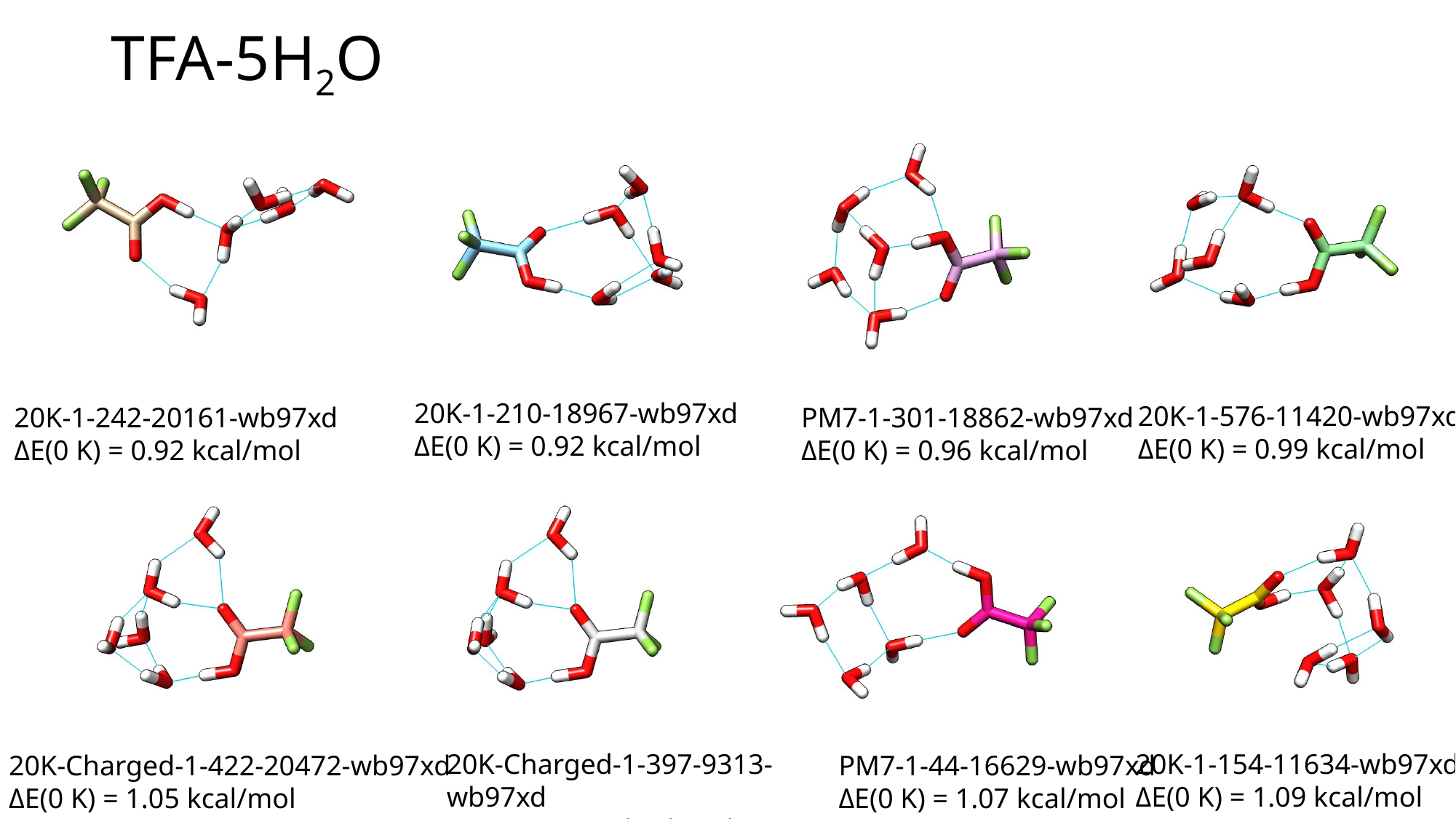

TFA-5H2O
20K-1-210-18967-wb97xd
ΔE(0 K) = 0.92 kcal/mol
20K-1-576-11420-wb97xd
ΔE(0 K) = 0.99 kcal/mol
PM7-1-301-18862-wb97xd
ΔE(0 K) = 0.96 kcal/mol
20K-1-242-20161-wb97xd
ΔE(0 K) = 0.92 kcal/mol
20K-Charged-1-397-9313-wb97xd
ΔE(0 K) = 1.06 kcal/mol
20K-1-154-11634-wb97xd
ΔE(0 K) = 1.09 kcal/mol
PM7-1-44-16629-wb97xd
ΔE(0 K) = 1.07 kcal/mol
20K-Charged-1-422-20472-wb97xd
ΔE(0 K) = 1.05 kcal/mol

## Slide 14
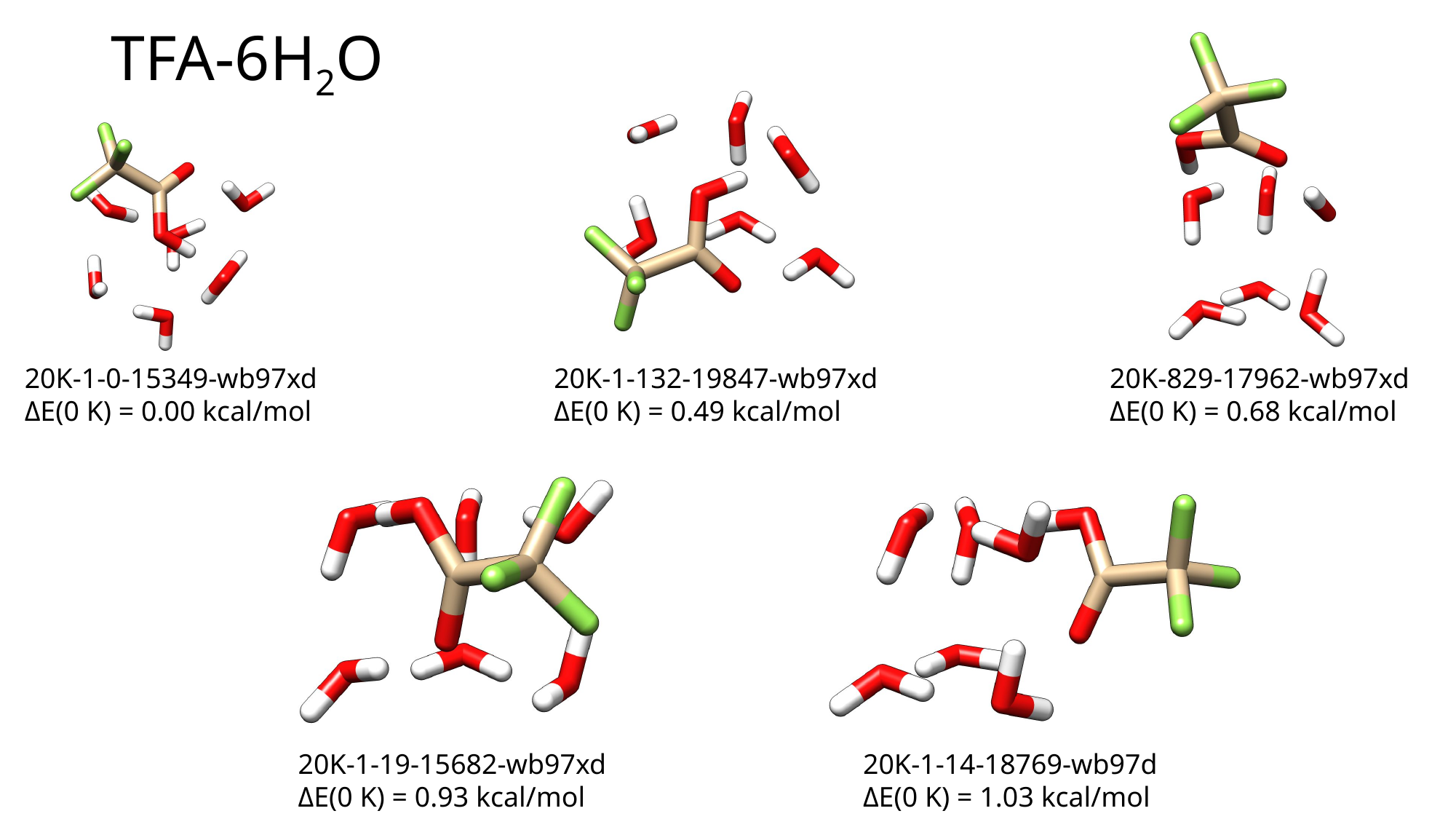

TFA-6H2O
20K-1-0-15349-wb97xd
ΔE(0 K) = 0.00 kcal/mol
20K-1-132-19847-wb97xd
ΔE(0 K) = 0.49 kcal/mol
20K-829-17962-wb97xd
ΔE(0 K) = 0.68 kcal/mol
20K-1-19-15682-wb97xd
ΔE(0 K) = 0.93 kcal/mol
20K-1-14-18769-wb97d
ΔE(0 K) = 1.03 kcal/mol

## Slide 15
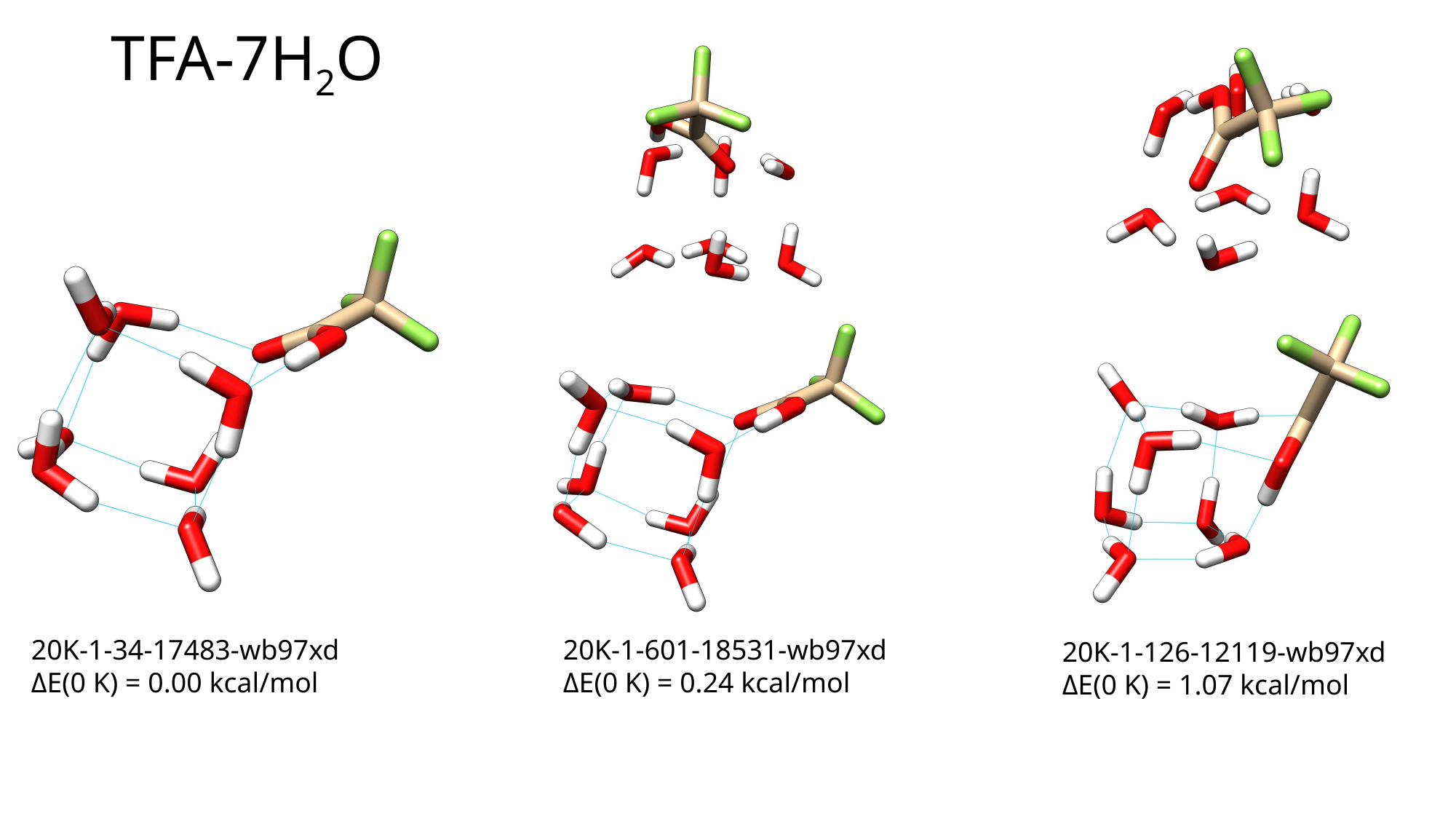

TFA-7H2O
20K-1-34-17483-wb97xd
ΔE(0 K) = 0.00 kcal/mol
20K-1-601-18531-wb97xd
ΔE(0 K) = 0.24 kcal/mol
20K-1-126-12119-wb97xd
ΔE(0 K) = 1.07 kcal/mol

## Slide 16
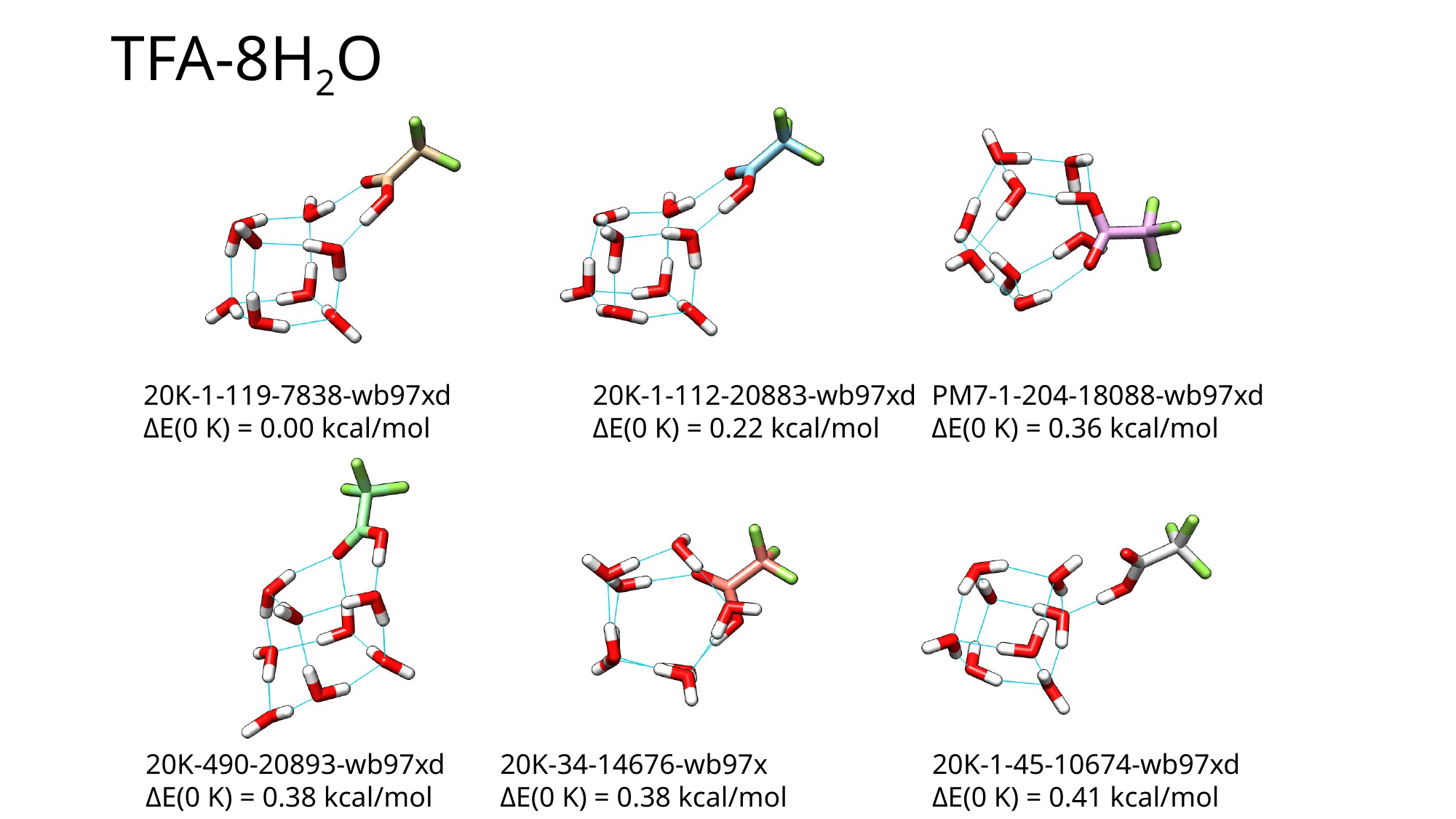

TFA-8H2O
20K-1-112-20883-wb97xd
ΔE(0 K) = 0.22 kcal/mol
PM7-1-204-18088-wb97xd
ΔE(0 K) = 0.36 kcal/mol
20K-1-119-7838-wb97xd
ΔE(0 K) = 0.00 kcal/mol
20K-34-14676-wb97x
ΔE(0 K) = 0.38 kcal/mol
20K-1-45-10674-wb97xd
ΔE(0 K) = 0.41 kcal/mol
20K-490-20893-wb97xd
ΔE(0 K) = 0.38 kcal/mol

## Slide 17
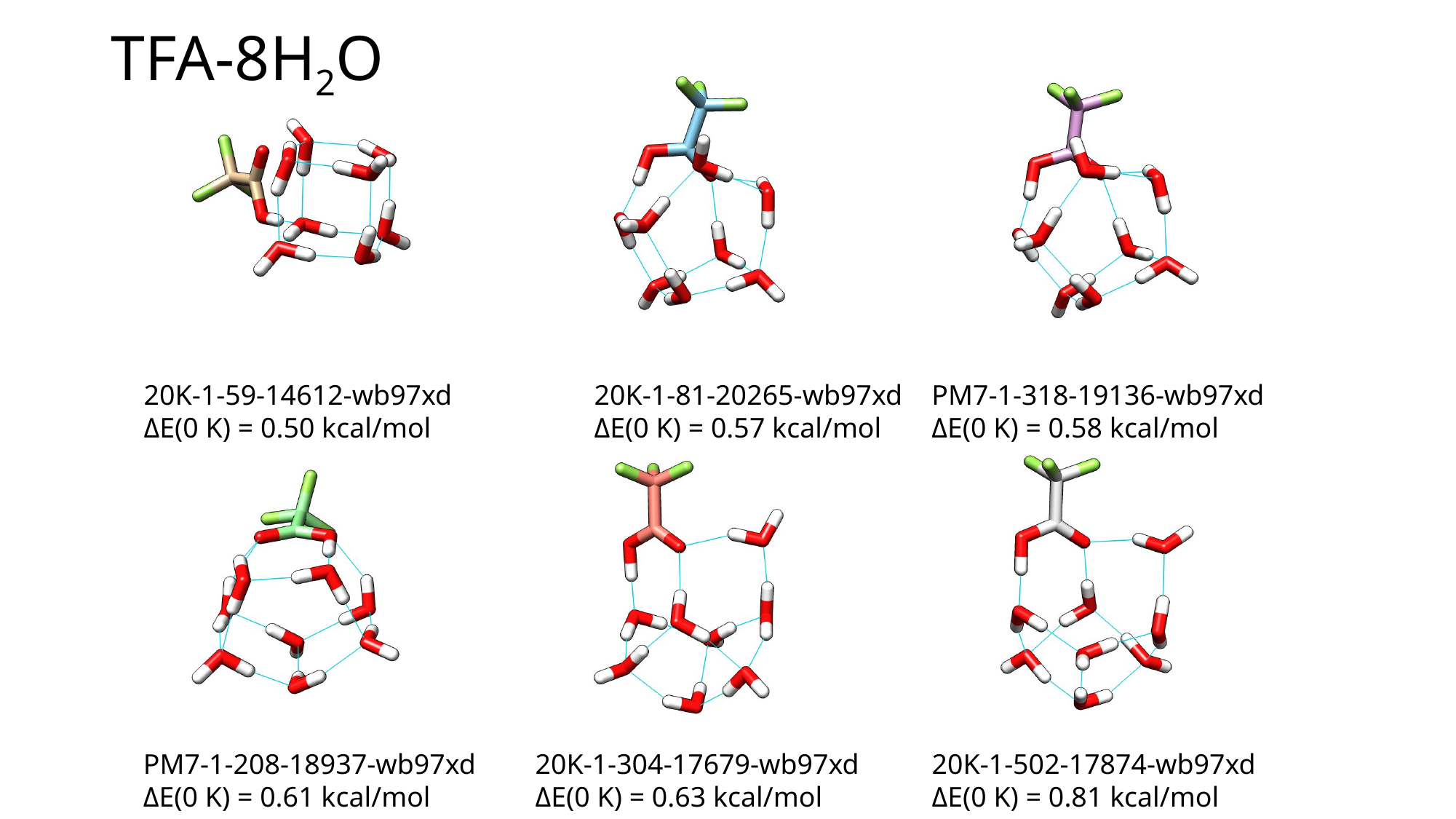

TFA-8H2O
20K-1-81-20265-wb97xd
ΔE(0 K) = 0.57 kcal/mol
PM7-1-318-19136-wb97xd
ΔE(0 K) = 0.58 kcal/mol
20K-1-59-14612-wb97xd
ΔE(0 K) = 0.50 kcal/mol
20K-1-304-17679-wb97xd
ΔE(0 K) = 0.63 kcal/mol
20K-1-502-17874-wb97xd
ΔE(0 K) = 0.81 kcal/mol
PM7-1-208-18937-wb97xd
ΔE(0 K) = 0.61 kcal/mol

## Slide 18
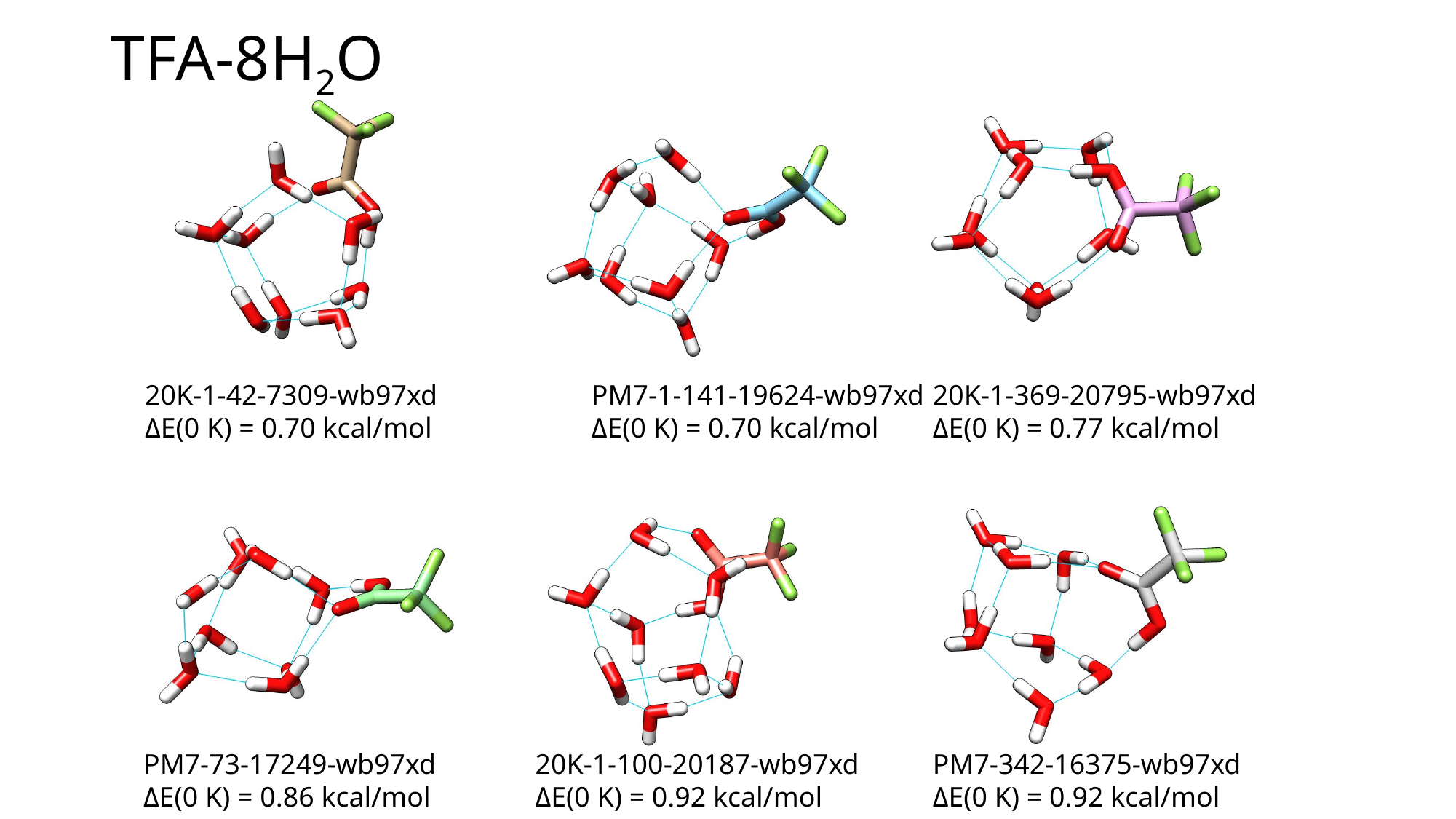

TFA-8H2O
PM7-1-141-19624-wb97xd
ΔE(0 K) = 0.70 kcal/mol
20K-1-369-20795-wb97xd
ΔE(0 K) = 0.77 kcal/mol
20K-1-42-7309-wb97xd
ΔE(0 K) = 0.70 kcal/mol
20K-1-100-20187-wb97xd
ΔE(0 K) = 0.92 kcal/mol
PM7-342-16375-wb97xd
ΔE(0 K) = 0.92 kcal/mol
PM7-73-17249-wb97xd
ΔE(0 K) = 0.86 kcal/mol

## Slide 19
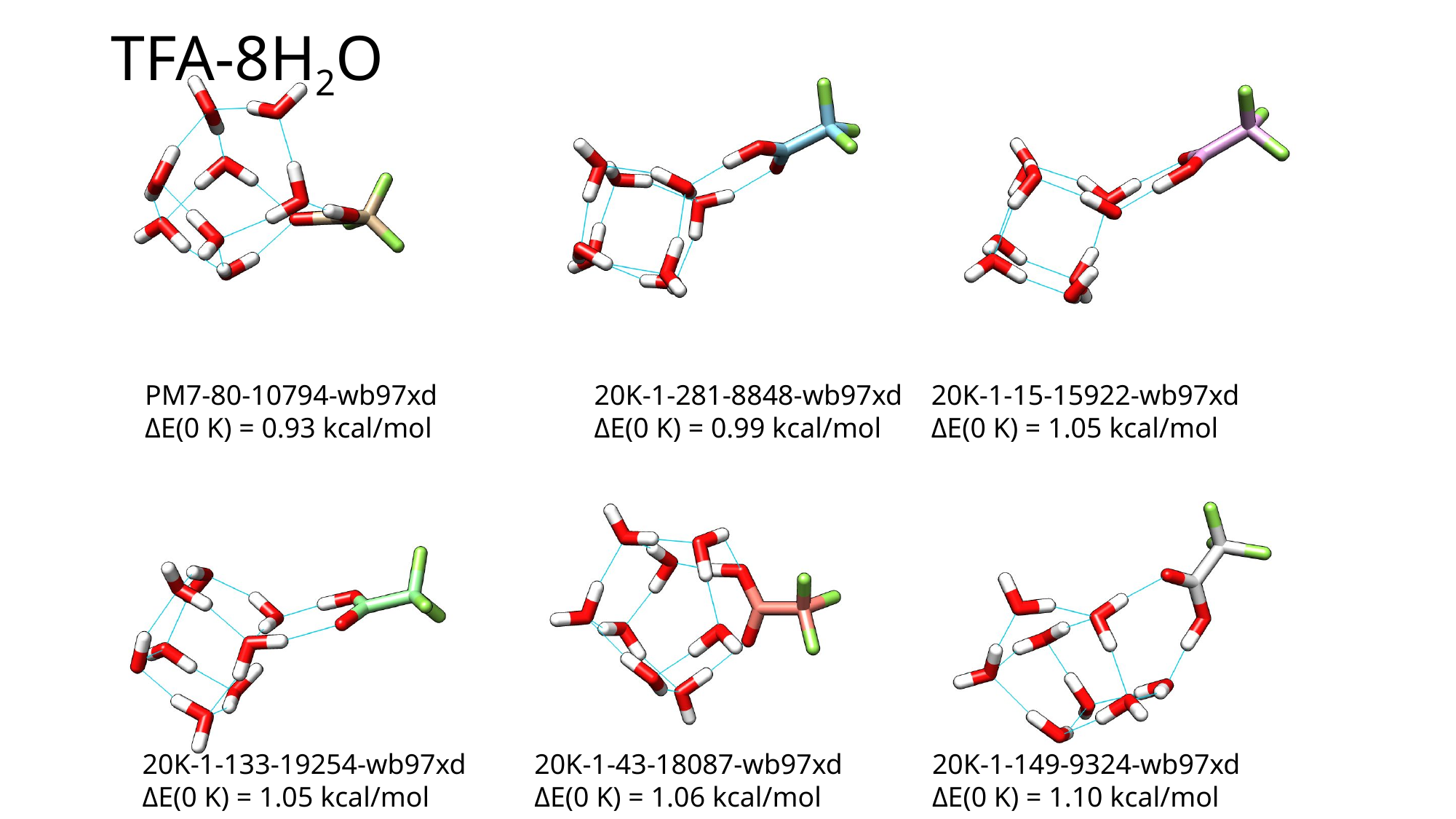

TFA-8H2O
20K-1-281-8848-wb97xd
ΔE(0 K) = 0.99 kcal/mol
20K-1-15-15922-wb97xd
ΔE(0 K) = 1.05 kcal/mol
PM7-80-10794-wb97xd
ΔE(0 K) = 0.93 kcal/mol
20K-1-43-18087-wb97xd
ΔE(0 K) = 1.06 kcal/mol
20K-1-149-9324-wb97xd
ΔE(0 K) = 1.10 kcal/mol
20K-1-133-19254-wb97xd
ΔE(0 K) = 1.05 kcal/mol
