# Supplementary material for: The Hydration of Trifluoroacetic Acid from 0 to 298 K
Source: J Phys Chem A. 2026 Jan 20;130(4):927–35. doi: 10.1021/acs.jpca.5c08151 (PMC12862820; doi:10.1021/acs.jpca.5c08151)
Supplement: Supplementary file 2 [file jp5c08151_si_002.pdf]

Supporting Information for

The Hydration of Trifluoroacetic Acid from 0 to 298 K

Walker J. Smith, Caroline S. Glick, George C. Shields*

Furman University, Department of Chemistry, 3300 Poinsett Hwy, Greenville, SC 29613, USA

Email: george.shields@furman.edu

Page Contents

S2 Analysis of NormalPNO and TightPNO Cutoffs
Figure S1.
Figure S2.
S3 ωB97X-D vs DLPNO-CCSD(T) Relative Energies
Figure S3.
Basis Set Convergence for DLPNO-CCSD(T)/haug-cc-pNVZ
Table S1.
S4 Experimental and Theoretical Spectroscopic Results for TFA-nH_2O
Table S2.
S5 Gibbs Free Energies of Formation of TFA-nH_2O at Various Temperatures
Table S3.
Table S4.
S6 Predicted Concentrations of TFA-nH_2O in the Atmosphere
Table S5.

1. Analysis of NormalPNO and TightPNO Cutoffs

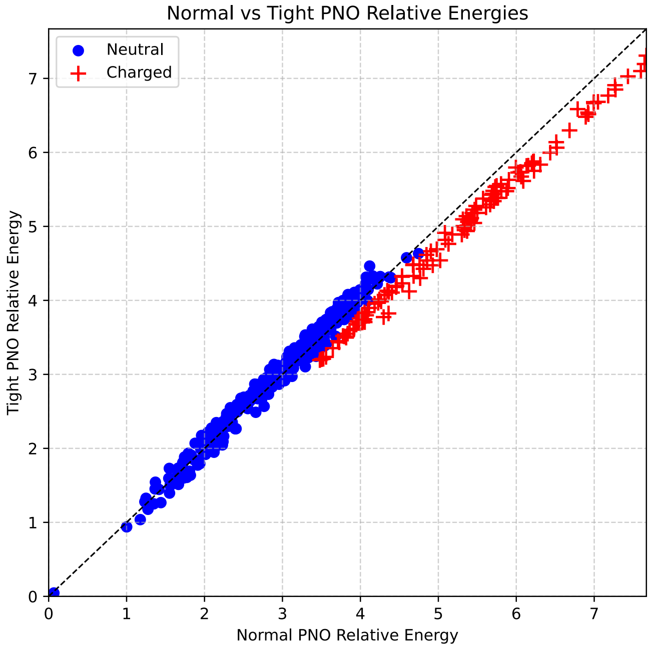

Figure S1. TightPNO vs NormalPNO DLPNO-CCSD(T)/haug-cc-pVQZ relative electronic energies of TFA-5H_2O isomers. Relative energies are computed with DLPNO-CCSD(T)/haug-cc-pV5Z and are presented in kcal/mol. Blue dots correspond to neutral clusters and red plus signs correspond to ion-pair clusters.

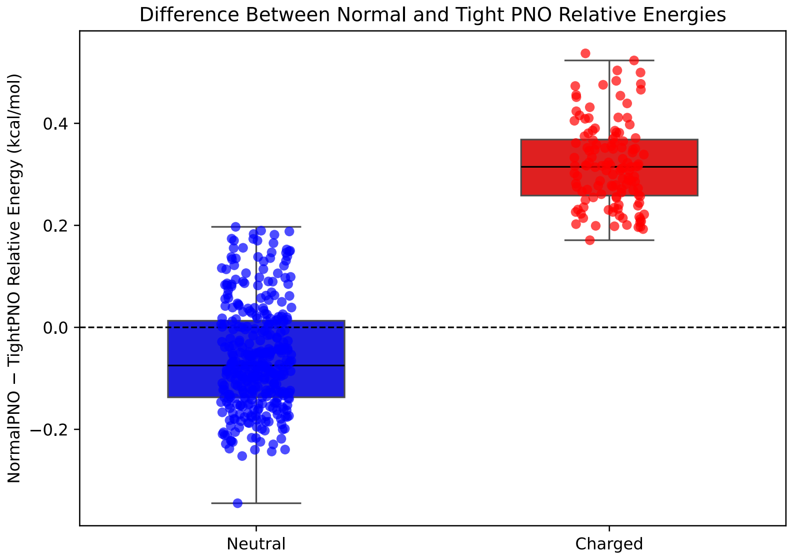

Figure S2. The error in NormalPNO relative to TightPNO relative electronic energies of TFA-5H_2O conformations using DLPNO-CCSD(T)/haug-cc-pVQZ model chemistry. Blue dots correspond to neutral clusters and red dots correspond to ion-pair clusters.

2. ωB97X-D vs DLPNO-CCSD(T) Relative Energies

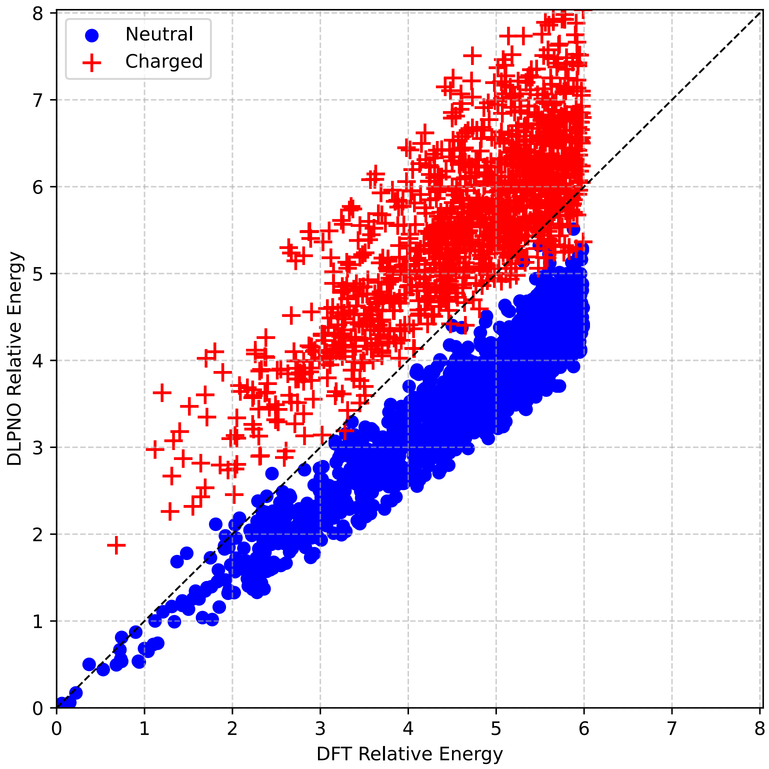

Figure S3. Relative energies (kcal/mol) of TFA-nH_2O (n=4-8) clusters with DLPNO-CCSD(T)/haug-cc-pV5Z versus ωB97X-D/6-31++G**.

3. Basis set convergence for DLPNO-CCSD(T)/haug-cc-pNVZ

Table S1. Mean absolute errors of DLPNO-CCSD(T)/haug-cc-pVNZ relative energies with various zeta-levels, N. Errors are in kcal/mol.

	D to T	T to Q	Q to 5	5 to 6
TFA-H_2O	0.263	0.116	0.032	0.019
TFA-2H_2O	0.337	0.053	0.025	0.013
TFA-3H_2O	0.189	0.070	0.033	0.015
TFA-4H_2O	0.259	0.093	0.047	0.017
TFA-5H_2O	0.372	0.149	0.058	N/A
TFA-6H_2O	0.746	0.265	0.106	N/A
TFA-7H_2O	0.384	0.231	0.083	N/A
TFA-8H_2O	0.469	0.335	0.085	N/A

4. Experimental and Theoretical Spectroscopic Results for TFA-nH2O

Table S2. Experimental and theoretical results for the lowest energy isomers of TFA-nH_2O, n=1-8, at 0 K. Rotational constants (A, B, C) and C=O stretching frequencies [ν(C=O)] from experiment,[1,2] previously published calculations,[1,2] and ωB97X-D/6-31++G**. The ωB97X-D/6-31++G** harmonic frequencies are scaled by 0.971. The previously published calculations of rotational constants used MP2/6-311++G** and vibrational frequencies used B971/6-311+G**.

	Expt	Prev. Calc.	ωB97X-D	% Error Prev. Calc.	% Error ωB97X-D
**TFA**					
A (MHz)	3865	3863	3852	-0.06%	-0.34%
B (MHz)	2499	2519	2485	0.81%	-0.54%
C (MHz)	2075	2083	2064	0.38%	-0.54%
ν(C=O) (cm^{-1})	1819	1871	1856	2.86%	2.06%
**TFA-H_2O**					
A	3835	3828/3830	3816	-0.19%/-0.13%	-0.49%
B	1083	1088/1106	1091	0.50%/2.16%	0.78%
C	994	997/1012	1000	0.32%/1.83%	0.66%
ν(C=O)	1793	1829	1873	2.01%	1.44%
**TFA-2H_2O**					
A	2534	2531/2561	2560	-0.11%/1.08%	1.03%
B	718	718/730	725	-0.06%/1.61%	0.95%
C	623	622/633	630	-0.08%/1.69%	1.14%
ν(C=O)	1767	1810	1801	2.43%	1.90%
**TFA-3H_2O**					
A	1733	1721/1752	1780	-0.71%/1.08%	2.69%
B	493	490/500	493	-0.69%/1.34%	-0.05%
C	415	410/419	416	-1.15%/1.02%	0.41%
ν(C=O)	1759	1814	1802	3.13%	2.45%
	**TFA-4H_2O**	**TFA-5H_2O**	**TFA-6H_2O**	**TFA-7H_2O**	**TFA-8H_2O**
A Calc	1380	1448	773	734	687
B Calc	368	262	412	243	170
C Calc	345	259	362	237	164
ν(C=O) Calc	1770	1794	1789	1752	1835
ν(C=O) Expt	1723, 1765	1728	1737		

5. Gibbs Free Energies of Formation of TFA-nH_2O at Various Temperatures

Table S3. Gibbs free energies of formation for TFA-nH_2O, n=1-8, at 0 K, 216.65 K, 273.15 K, and 298.15 K. Gibbs free energies are computed with DLPNO-CCSD(T)/haug-cc-pV5Z//ωB97X-D/6-31++G**.

	0 K	216.65 K	273.15 K	298.15 K
TFA-H_2O	-8.60	-2.64	-0.94	-0.19
TFA-2H_2O	-17.18	-5.40	-2.01	-0.52
TFA-3H_2O	-23.68	-6.61	-1.77	0.33
TFA-4H_2O	-30.52	-7.40	-1.05	1.72
TFA-5H_2O	-37.07	-8.84	-0.34	3.24
TFA-6H_2O	-45.75	-9.10	1.07	5.55
TFA-7H_2O	-54.46	-10.48	2.17	7.69
TFA-8H_2O	-61.66	-12.06	2.34	8.69

Table S4. Gibbs free energies of formation for the lowest-energy ion-pair structures of TFA-nH_2O, n=1-8, at 0 K. Gibbs free energies are computed with DLPNO-CCSD(T)/haug-cc-pV5Z//ωB97X-D/6-31++G**.

	0 K
TFA-H_2O	n/a
TFA-2H_2O	n/a
TFA-3H_2O	n/a
TFA-4H_2O	-27.96
TFA-5H_2O	-35.13
TFA-6H_2O	-43.05
TFA-7H_2O	-52.06
TFA-8H_2O	-60.32

6. Predicted Concentrations of TFA-nH2O in the Atmosphere

Table S5. Equilibrium concentrations of clusters at 216.65 K and 298.15 K. Initial concentrations of monomers at 217 K were TFA = 5.0 x 10^6 cm^{-3} and H_2O = 9.90 x 10^{17} cm^{-3}. Initial concentrations of the monomers at 298 K were TFA = 5.0 x 10^3 cm^{-3} and H_2O = 7.7x10^{14} cm^{-3}.

	216.65 K	298.15 K
TFA	4.93e3	4.78e6
H_2O	9.90e14	7.7e17
TFA-H_2O	6.64e1	2.06e5
TFA-2H_2O	1.18	1.13e4
TFA-3H_2O	5.74e-4	8.40e1
TFA-4H_2O	1.05e-7	2.52e-1
TFA-5H_2O	3.77e-11	6.05e-4
TFA-6H_2O	4.66e-15	3.84e-7
TFA-7H_2O	3.36e-18	3.24e-10
TFA-8H_2O	3.85e-21	1.88e-12

References

1. Ouyang, B.; Starkey, T. G.; Howard, B. J. High-Resolution Microwave Studies of Ring-Structured Complexes between Trifluoroacetic Acid and Water. *The Journal of Physical Chemistry A* **2007**, *111* (28), 6165–6175.
2. Ito, F. Stable isomers for trifluoroacetic acid (TFA) pentahydrates obtained from density functional calculations. *Vibrational Spectroscopy* **2014**, *71*, 57–61.
